# Lactylation of HDAC1 Confers Resistance to Ferroptosis in Colorectal Cancer

**DOI:** 10.1002/advs.202408845

**Published:** 2025-01-31

**Authors:** Zhou Yang, Wei Su, Qinglin Zhang, Lili Niu, Baijie Feng, Yu Zhang, Feng Huang, Jiaxin He, Qinyao Zhou, Xin Zhou, Longjun Ma, Jingwan Zhou, Yuanrong Wang, Wenjing Xiong, Jun Xiang, Zhilin Hu, Qiang Zhan, Bing Yao

**Affiliations:** ^1^ Department of Head and Neck Surgery Fudan University Shanghai Cancer Center Shanghai 200032 China; ^2^ Department of Oncology Shanghai Medical College Fudan University Shanghai 200032 China; ^3^ Department of Medical Oncology Fudan University Shanghai Cancer Center Shanghai 200032 China; ^4^ Departments of Gastroenterology Wuxi People's Hospital Affiliated to Nanjing Medical University Nanjing Medical University Nanjing Jiangsu 214043 China; ^5^ Department of Integrative Medicine Shanghai Pulmonary Hospital Tongji University Medical School Cancer Institute Tongji University School of Medicine Shanghai 200433 China; ^6^ Department of Medical Oncology Shanghai Pudong Hospital Fudan University Pudong Medical Center Shanghai 201399 China; ^7^ Department of General Surgery, The Affiliated Taizhou People's Hospital of Nanjing Medical University, Taizhou School of Clinical Medicine; National Experimental Teaching Center of Basic Medical Science, Department of Medical Genetics School of Basic Medical Sciences Nanjing Medical University Nanjing 211166 China; ^8^ Department of Epidemiology School of Public Health Nanjing Medical University Nanjing 211166 China; ^9^ Department of Immunology Key Laboratory of Immune Microenvironment and Disease The School of Basic Medicine; Department of laboratory medicine, the first affiliated hospital of Nanjing Medical University Nanjing Medical University Nanjing 211166 China; ^10^ State Key Laboratory Cultivation Base of Biomarkers for Cancer Precision Prevention and Treatment, Collaborative Innovation Center for Cancer Personalized Medicine; NHC Key Laboratory of Antibody Technique, Jiangsu Province Engineering Research Center of Antibody Drug Nanjing Medical University Nanjing 211166 China

**Keywords:** colorectal cancer, ferroptosis, HDAC inhibitor, lactylation, N6‐methyladenosine modification

## Abstract

Colorectal cancer (CRC) is highly resistant to ferroptosis, which hinders the application of anti‐ferroptosis therapy. Through drug screening, it is found that histone deacetylase inhibitor (HDACi) significantly sensitized CRC to ferroptosis. The combination of HDACi and ferroptosis inducers synergically suppresses CRC growth both in vivo and in vitro. Mechanically, HDACi reduces ferroptosis suppressor protein (FSP1) by promoting its mRNA degradation. Specifically, it is confirmed that HDACi specifically targets HDAC1 and promotes the H3K27ac modification of fat mass‐ and obesity‐associated gene (FTO) and AlkB Homolog 5, RNA Demethylase (ALKBH5), which results in significant activation of FTO and ALKBH5. The activation of FTO and ALKBH5 reduces N6‐methyladenosine (m^6^A) modification on FSP1 mRNA, leading to its degradation. Crucially, lactylation of HDAC1^K412^ is essential for ferroptosis regulation. Both Vorinostat (SAHA) and Trichostatin A (TSA) notably diminish HDAC1^K412^ lactylation in comparison to other HDAC1 inhibitors, exhibiting a consistent trend of increasing susceptibility to ferroptosis. In conclusion, the research reveals that HDACi decreases HDAC1^K412^ lactylation to sensitize CRC to ferroptosis and that the combination of HDACi and ferroptosis inducers can be a promising therapeutic strategy for CRC.

## Graphical abstract

HDAC inhibitor decreases HDAC1^K412^ lactylation to sensitize colorectal cancer to ferroptosis and the combination of HDAC inhibitor and ferroptosis inducers can be a promising therapeutic strategy for colorectal cancer.

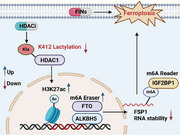

## 1 Introduction

Colorectal cancer (CRC) looms large as a malignancy that poses a serious threat to human health. Despite advancements, tumor recurrence and metastasis persist as principal contributors to postoperative mortality, and are pivotal in impeding considerable progress toward enhancing patient survival and prognosis.^[^ ^1^ ^]^ Consequently, the exploration of efficacious treatment modalities to counteract recurrence and metastasis is paramount to fostering continued improvements in patient survival and prognosis.

Ferroptosis, an iron‐dependent form of regulated cell death driven by excessive lipid peroxides on cell membranes, stands distinct from apoptosis and other established regulatory cell death mechanisms.^[^ ^2^ ^]^ Indeed, current strides underline the promising utility of ferroptosis inducers, alone or in combination with radiotherapy, chemotherapy, and immunotherapy, underscoring its importance as a pioneering strategy in contemporary tumor treatment. The appreciable therapeutic efficacy of ferroptosis has been substantiated, with select cancer cells, such as clear cell renal cell carcinoma (ccRCC), small cell lung cancer (SCLC), and triple‐negative breast cancer (TNBC), demonstrating vulnerability owing to their distinctive metabolic attributes, including elevated polyunsaturated fatty acid (PUFA) ether phospholipids or unstable intracellular iron pools.^[^ ^3^ ^]^ Nevertheless, the strong resistance of CRC, characterized by its exceptional malignancy, poses a formidable impediment to the clinical implementation of ferroptosis therapy.^[^ ^4^ ^]^

Histone deacetylase (HDAC) wraps DNA tightly around histones, making them less susceptible to contact by gene transcription factors. The tumor‐promoting effect of HDAC is mediated through various mechanisms, including transcriptional suppression and gene silencing, regulation of cell cycle progression, modulation of cell apoptosis, upregulation of angiogenesis, promotion of tumor invasion and metastasis, as well as facilitation of immune escape. HDAC inhibitors (HDACi) have garnered attention owing to their inhibitory impact on essential tumor cell functions such as migration, invasion, and metastasis, consequently becoming a seminal area of investigation.^[^ ^5^ ^]^ Increasingly, evidence suggests that HDACi not only exhibits standalone efficacy but also demonstrates a capacity to augment the effectiveness of various other treatment modalities. Notably, in CRC, the pan‐HDACi vorinostat (SAHA) sensitizes CRC to radiation therapy and amplifies the combinatorial efficacy of capecitabine‐based radiotherapy and chemotherapy regimen.^[^ ^6^ ^]^ Although HDACi have exhibited efficacy in extending patient survival in CRC, their dose‐dependent toxicity, stands as a significant adverse effect that curtails their broader application.^[^ ^7^ ^]^ These side effects include: hematologic toxicity,^[^ ^8^ ^]^ gastrointestinal toxicity,^[^ ^9^ ^]^ cardiovascular toxicity,^[^ ^10^ ^]^ metabolic disturbances,^[^ ^11^ ^]^ fatigue and weakness,^[^ ^12^ ^]^ and immune‐related side effects.^[^ ^13^ ^]^ It is noteworthy that an increasing body of research demonstrates the efficacy of combining low doses of HDACi with other medications to address the issue of adverse effects.^[^ ^14^ ^]^ Consequently, a pivotal need arises to recalibrate the dose of HDACi and explore their combination with other drugs to elicit antitumor effects at safer levels for advancing CRC treatment.

Aberrant metabolism stands as a hallmark of cancer.^[^ ^15^ ^]^ Despite the presence of oxygen, a majority of cancer cells rely on aerobic glycolysis—a phenomenon known as the “Warburg effect.”^[^ ^16^ ^]^ This metabolic shift primes the microenvironment, resulting in heightened levels of glycolytic intermediates such as lactate, thereby facilitating cell proliferation.^[^ ^17^ ^]^ Recently, the identification of lactylation as a novel posttranslational modification in both histones and nonhistone proteins has provided a new outlook on the nonmetabolic functions of lactate.^[^ ^18^ ^]^ While protein lactylation has garnered increasing recognition as a prevalent posttranslational modification, particularly in tumor cells, the specific enzyme responsible for catalyzing existing articles has solely indicated that HADC1‐3 function as histone lysine deacetylases.^[^ ^19^ ^]^ There is currently no research available on whether members of the HDAC family undergo lactylation modification themselves or if members of HDACi are involved in non‐histone deacetylation. Understanding the mechanisms underlying HDACi regulation may broaden the therapeutic strategies to inhibit HDAC activity in patients with cancer.

In this study, we demonstrated that HDACi sensitized CRC to ferroptosis by repressing ferroptosis suppressor protein (FSP1), a known ferroptosis defender overexpressed in CRC. By unraveling this mechanism, we revealed the intricate interplay between histone modifications and RNA methylation modifications. The diminishment of HDAC1^K412^ lactylation by HDACi is crucial for regulating ferroptosis. Together, these studies expand the fundamental knowledge urgently needed for how HDACi decreases HDAC1 lactylation and identify that the combination of HDACi and ferroptosis inducer (FIN) treatment may be an innovative and potent therapeutic strategy for CRC.

## 2 Results

### 2.1 HDACi Confers Targetable Potential of CRC to Ferroptosis

**Figure 1:**
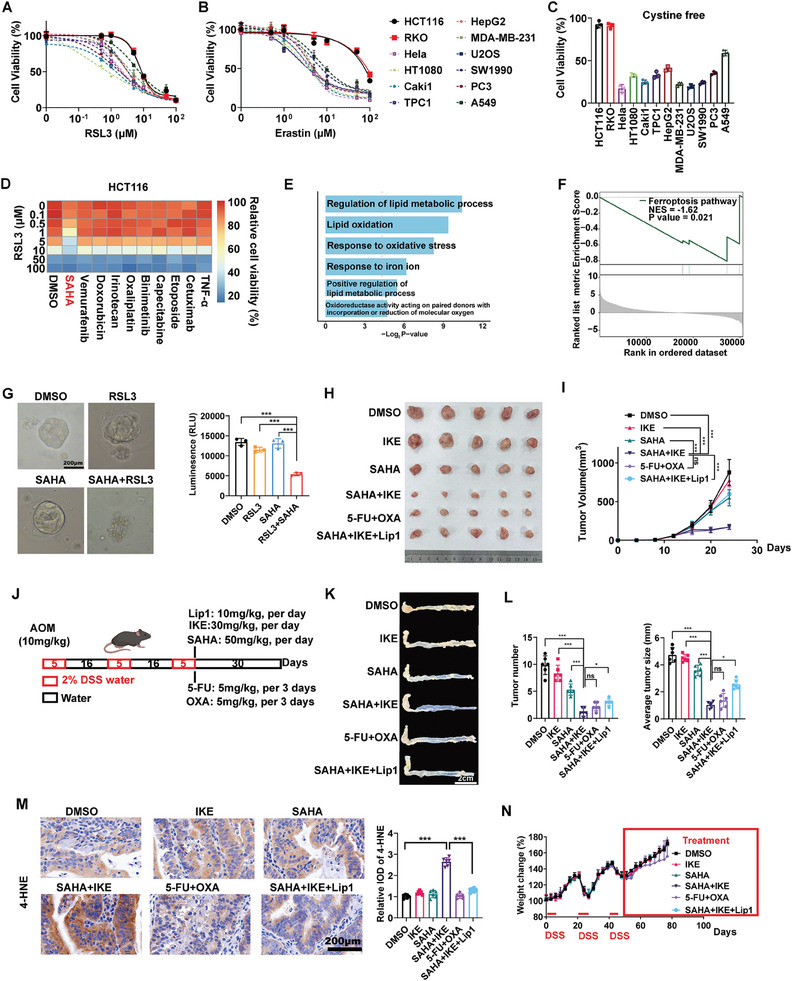
HDACi confers the targetable potential of CRC to ferroptosis. A–C) The response of different cancer cell lines to ferroptosis inducers (24 h): RSL3 (A), erastin (B), cystine starvation C). D) Sublethal dose (lethal concentration/LC10‐30) of ferroptosis inducer RSL3 (5 µm) combined with multiple sublethal doses of chemotherapy (doxorubicin: 0.01 µm; irinotecan: 1 µm; oxaliplatin: 5 µm; capecitabine: 5 µm; etoposide: 50 µm), targeted therapy (vemurafenib: 1 µm; SAHA: 1 µm; binimetinib: 5 µm; cetuximab: 100 µg mL^−1^), and immunotherapy drugs (TNF‐α: 100 ng mL^−1^). Detailed dose‐response curves of each drug were provided in Figure S1 (Supporting Information). E–F) Gene Ontology (GO) analysis (E) and GSEA (F) of differentially expressed genes between HCT116 cells treated with or without SAHA (1 µm, 24 h). G) Morphological characterization and viability of intestinal organoids treated with SAHA (1 µm) and RLS3 (5 µm) for 24 h. Quantitative detection of intestinal organoid activity (*n* = 3 organoids per group). H,I) Representative images (H), and tumor volume (I) of HCT116‐derived xenografts. IKE (30 mg kg^−1^, i.p., per day), SAHA (50 mg kg^−1^, i.p., per day), 5‐FU (5 mg kg^−1^, i.p., per 3 days), OXA (5 mg kg^−1^, i.p., per 3 days), Lip1 (10 mg kg^−1^, i.p., per day). J) Construction of AOM/DSS induced CRC model and treatment strategy. K,L) Representative images of colons (K), quantifications of tumor number and size (L), and IHC staining of 4‐HNE (ferroptosis index) M), and weight change curve N) in AOM/DSS induced CRC model. IHC was quantified by integrated optical density (IOD). (*ns*: no significance, ^*^ *p* < 0.05, ^***^ *p* < 0.001).

We induced ferroptosis in various tumor cell lines through various strategies using RSL3, erastin, and cystine starvation. Interestingly, our findings revealed that the CRC cell lines (HCT116, RKO) demonstrated substantial resistance to ferroptosis induced by these strategies (**Figure** **1**A–C). Given the close relationship demonstrated in previous studies between ferroptosis and the sensitivity of chemotherapy, targeted therapy, and immunotherapy, we were intrigued by the possibility that these therapies may also reverse‐regulate the sensitivity of ferroptosis inducers.^[^ ^3^, ^20^ ^]^ Following the induction of ferroptosis with RSL3, we combined multiple sublethal doses (LC10‐30) of the ferroptosis inducer RSL3 (2.5 µm) with various chemotherapies (doxorubicin, irinotecan, oxaliplatin, capecitabine, etoposide), targeted therapies (vemurafenib, SAHA, binimetinib, cetuximab), and immunotherapy drugs (TNF‐α) to identify agents that may enhance the sensitivity of CRC to ferroptosis. Among these, the histone deacetylase inhibitor (HDACi), vorinostat (SAHA), demonstrated a remarkably potent sensitization effect toward ferroptosis (Figure 1D; Figure S1, Supporting Information). Following RNA‐seq analysis of SAHA‐treated HCT116 cells, we discovered that SAHA treatment is linked to several important pathways involved in ferroptosis, including lipid metabolic processes, lipid peroxidation, and response to iron ions (Figure 1E). Additionally, further Gene Set Enrichment Analysis (GSEA) also validated a significant association between the ferroptosis pathway and SAHA treatment (Figure 1F). Upon further analysis, we confirmed that SAHA and RSL3 synergistically reduced the viability of intestinal organoids (Figure 1G). In combination, these findings affirm that SAHA sensitizes ferroptosis through an unknown mechanism in vitro.

Furthermore, we validated that sublethal concentrations of SAHA enhanced the lethal effect of multiple doses of RSL3 in four different CRC cell lines (HCT116, RKO, SW620, and HT29) (Figure S2A–D, Supporting Information). We then delved into whether SAHA also mediates ferroptosis induced by mechanisms distinct from RSL3‐mediated GPX4 inhibition. To investigate this, we selected a series of small molecules targeting various ferroptosis‐related factors with dissimilar structures. These chemicals included ML210 (another inhibitor of GPX4), erastin – an inhibitor of solute carrier family 7 member 11 (SLC7A11), FINO_2_– a 1,2‐dioxolane that directly oxidizes iron and indirectly inhibits GPX4 enzymatic function, and direct cystine starvation. We found that SAHA also promoted the lethal effect of other FINs in HCT116, including erastin, ML210, and FINO_2_ (Figure S2E–H, Supporting Information). Furthermore, SAHA promoted cell death and lipid peroxidation induced by FINs: RSL3, erastin, and FINO_2_ and cystine starvation in HCT116. Remarkably, ferroptosis inhibitor deferoxamine (DFO) significantly rescued promoted cell death and lipid peroxidation induced by different FINs (Figure S2I–K, Supporting Information). Reduced cell viability due to lethal dose SAHA could be rescued by the necrosis inhibitor necrostatin‐1 and apoptosis inhibitor Z‐VAD‐FMK, but not DFO, which indicated that SAHA induced a mix of necrosis and apoptosis, but not ferroptosis (Figure S2L, Supporting Information). However, in the combined therapy (sublethal SAHA+RSL3), only DFO rescued inhibited cell viability (Figure S2 m, Supporting Information). Taking these data together, we confirmed that SAHA sensitized ferroptosis, but not that FINs sensitized SAHA. As SAHA is a pan‐HDACi, we further investigated whether other types of HDACi sensitized FINs in CRC cells, including TSA and SB. All sublethal doses of HDACi promoted ferroptosis induced by RSL3 (Figure S2N–P, Supporting Information). Overall, HDACi represented by SAHA confers the targetable potential of CRC cells to ferroptosis.

We next investigated whether SAHA‐sensitized ferroptosis was induced by IKE, a metabolically stable erastin analog in vivo. As expected, IKE alone failed to reduce CRC growth, whereas the combination of SAHA and IKE synergically repressed tumor growth. Additional treatment of ferroptosis inhibitor Lip1 alleviated the repression effect of combination therapy, indicating that SAHA specifically sensitized ferroptosis in vivo. Meanwhile, the effect of SAHA+IKE was similar to that of standard chemotherapy of CRC (5‐fluorouracil+oxaliplatin, 5‐FU+OXA) (Figure 1H,I). Subsequently, we constructed an AOM/DSS orthotopic CRC model to assess the effectiveness and safety of combination therapy in a real CRC tumor microenvironment (Figure 1J). In line with that of the xenografts model, SAHA and IKE synergically induced ferroptosis in CRC and repressed tumor growth (Figure 1K,L). Additionally, SAHA+IKE significantly increased 4‐Hydroxynonenal (4‐HNE) expression (index of ferroptosis) compared to a single treatment alone (Figure 1M). Notably, 2/6 mice in the standard chemotherapy group showed body decreased weight (Figure 1N). Although pathology examination showed no organ injury in each group, laboratory examination indicated a decrease in white blood cells (WBC), red blood cells (RBC), lymphocytes (LYM), platelets (PLT), and hemoglobin (HGB) in these 2 mice in the standard chemotherapy group (Figures S3 and S4, Supporting Information). These examinations indicated potential myelosuppression caused by long‐term standard chemotherapy, which is one of the most common adverse reactions in the clinical application of 5‐FU and OXA. Overall, the combination therapy involving SAHA and IKE presents a potential and promising approach for the treatment of CRC in vivo.

### 2.2 HDACi Drives Response to Ferroptosis Inducers by Repressing FSP1

**Figure 2:**
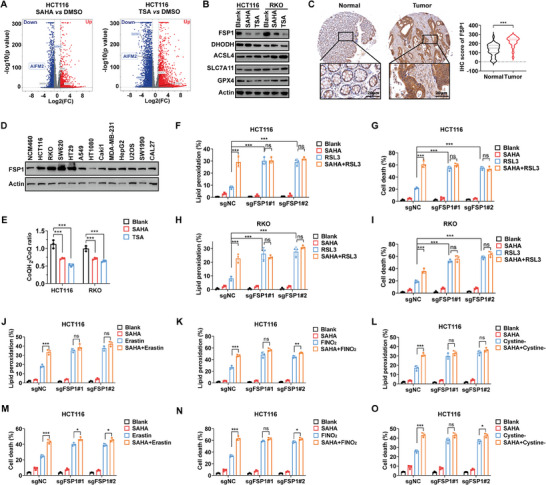
HDACi drives response to ferroptosis inducer by repressing FSP1. A) Volcano map showed differentially expressed genes of HCT116 treated with SAHA (1 µm) and TSA (0.1 µm) for 24 h. B) Western Blotting analysis revealed the expression of the main ferroptosis regulator of CRC cells treated with SAHA (1 µm) and TSA (0.1 µm) for 24 h. C) Expression of FSP1 in CRC and paired normal bowel tissues (n = 79). D) Expression of FSP1 in different cell lines detected by Western Blotting. E) Ratio of CoQH_2_/CoQ of CRC cells treated with SAHA (1 µm) and TSA (0.1 µm) for 24 h. F,G) Lipid peroxidation (F, detected at 12 h) and cell death (G, detected at 24 h) of FSP1‐depleted HCT116 cells treated with SAHA (1 µm) and RSL3 (5 µm). H,I) Lipid peroxidation (H, detected at 12 h) and cell death (I, detected at 24 h) of FSP1‐depleted RKO cells treated with SAHA (1 µm) and RSL3 (5 µm). J–L) Lipid peroxidation of FSP1‐depleted HCT116 cells treated with SAHA (1 µm) and different FINs, including erastin (J, 40 µm), FINO_2_ (K, 20 µm), and cystine starvation (L) for 12 h. M–O) Cell death of HCT116 cells treated with SAHA and different FINs, including erastin (M, 40 µm), FINO_2_ (N, 20 µm), and cystine starvation (O) for 24 h. (*ns*: no significance, ^*^ *p* < 0.05, ^**^ *p* < 0.01, ^***^ *p* < 0.001).

Through RNA‐seq and Western Blotting analysis, we screened main ferroptosis regulators, including FSP1 (*AIFM2*), SLC7A11, ACSL4, and more. Interestingly, both SAHA and TSA significantly reduced the expression of FSP1 (**Figure** **2**A,B). In line with the results in vitro, SAHA also significantly repressed FSP1 in the AOM/DSS‐induced CRC mouse model (Figure S5A, Supporting Information). Additionally, we confirmed that FSP1 is overexpressed in CRC compared with normal bowel tissues (Figure 2C). FSP1 was also overexpressed in CRC cell lines (HCT116, RKO, SW620, and HT29) compared with normal colonic epithelial cells (NCM460) and other cancer cell lines (HT1080, etc.) (Figure 2D). Overexpression of FSP1 in CRC interpreted its resistance to ferroptosis as described above (Figure 1). FSP1 reduces ubiquinone (CoQ) to ubiquinol (CoQH_2_) on the plasma membrane and inner mitochondrial membrane, respectively. CoQH_2_ can neutralize lipid peroxyl radicals as a radical‐trapping antioxidant, resulting in ferroptosis suppression.^[^ ^21^ ^]^ As expected, SAHA and TSA both potently reduced the ratio of CoQH_2_/CoQ (Figure 2E). Subsequently, we depleted FSP1 in CRC cell lines (Figure S5B, Supporting Information). Notably, in FSP1‐depleted CRC cells, the promotional effect of SAHA on RSL3‐induced lipid peroxidation and cell death was abolished (Figure 2F–I). Similarly, the sensitization of SAHA on other FINs was also completely abolished, including erastin, FINO_2_, and cystine starvation (Figure 2J–O). Meanwhile, overexpression of FSP1 in SAHA and RSL3‐treated CRC cells successfully rescued promoted ferroptosis (Figure S5C–G, Supporting Information). Taken together, we confirmed that FSP1 is the target for HDACi‐induced ferroptosis sensitivity.

### 2.3 HDACi Accelerates mRNA Degradation of FSP1 Dependent on m^6^A RNA Methylation

**Figure 3:**
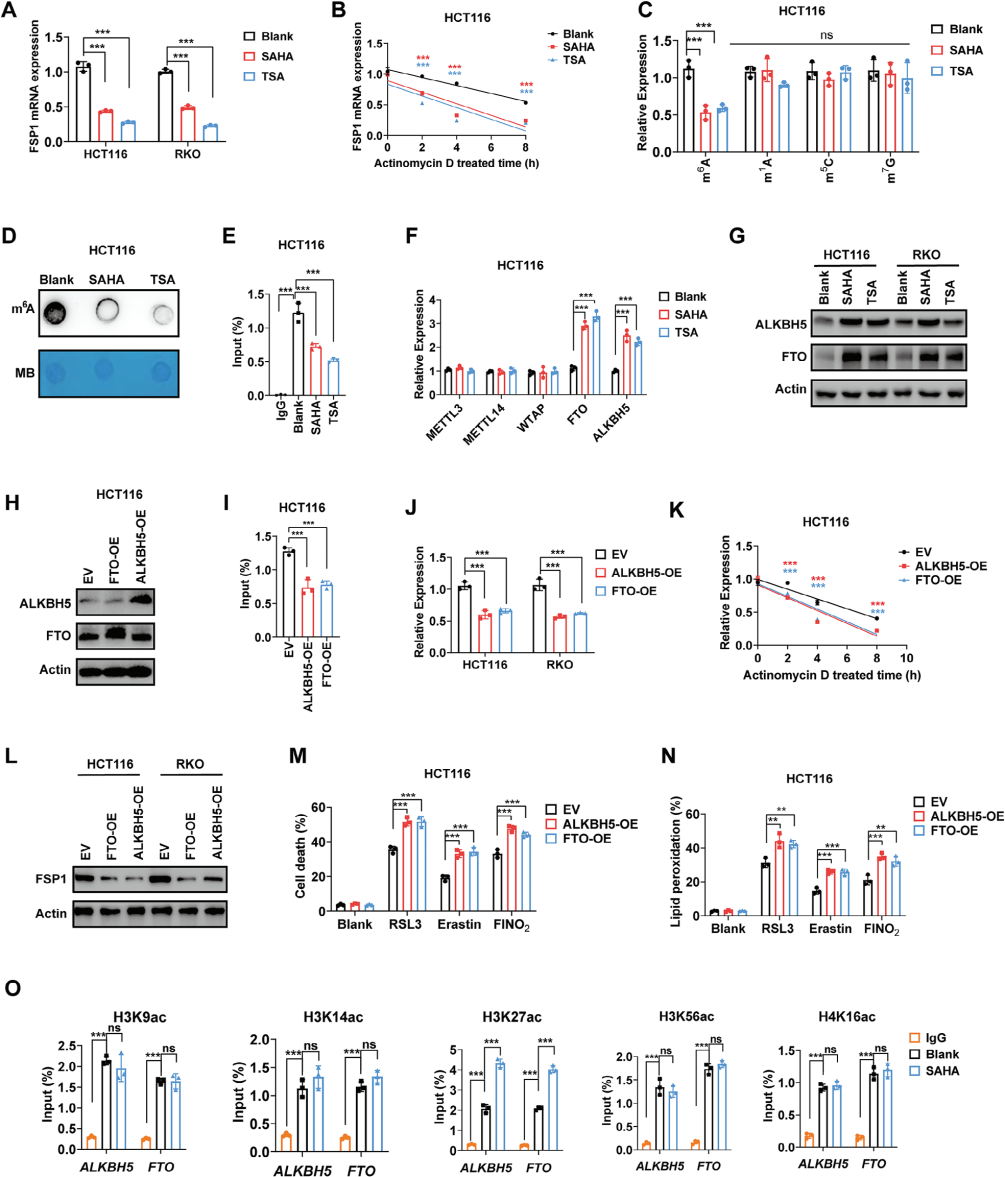
HDACi accelerates mRNA degradation of FSP1 dependent on m^6^A RNA methylation. A,B) mRNA expression (A) and stability (B) of FSP1 in CRC cells treated with SAHA (1 µm) and TSA (0.1 µm) for 24 h. C) Different nucleic acid modifications in CRC cells treated with SAHA (1 µm) and TSA (0.1 µm) for 24 h. N(6)‐methylation of adenosine: m^6^A; N(1)‐methylation of adenosine: m^1^A; 5‐methylcytosine: m^5^C; N7‐methylguanosine: m^7^G. D) m^6^A modification of total mRNA in HCT116 cells detected by Dot Blot. E) m^6^A level of FSP1 mRNA in HCT116 cells detected by meRIP‐qPCR. F,G) The mRNA (F) and protein (G) expression of m^6^A writers and erasers. H) Overexpression of ALKBH5 and FTO in HCT116 validated by Western Blotting analysis. I–L) Overexpression of ALKBH5 and FTO repressed m^6^A level (I), mRNA expression (J), mRNA stability (K), and protein expression (L) of FSP1. M,N) Cell death (M, detected at 24 h) and lipid peroxidation (N, detected at 12 h) of HCT116 cells treated with different FINs (RSL3: 5 µM, erastin: 40 µM, FINO_2_: 20 µM). O) Histon acetylation of *FTO* and *ALKBH5* in HCT116 cells treated with or without SAHA (1 µm, 24 h) detected by CHIP. (*ns*: no significance, ^**^ *p* < 0.01, ^***^ *p* < 0.001).

HDACi increases histone acetylation of its direct targets, thus promoting transcription output.^[^ ^22^ ^]^ However, in our study, HDACi repressed FSP1 expression, suggesting that FSP1 should not be the direct target of HDACi and other potential middle mechanisms should exist. We employed rMATS to identify potential alternative splicing events following HDACi treatment. As illustrated in the sashimi plot, although transcript levels were elevated, only skipping exon (SE) events and mutually exclusive exon (MXE) events were observed in the SAHA and TSA treated cells. These events were not statistically significant, with all *p*‐values exceeding 0.05 when compared to the DMSO group (Figure S6A, Supporting Information). Additionally, we predicted potential RNA‐Binding Protein (RBP) of FSP1 through the RNA‐Binding Protein Data Base (http://rbpdb.ccbr.utoronto.ca/).^[^ ^23^ ^]^ 22 potential RBPs of FSP1 were predicted and shown in Table S6 (Supporting Information). Subsequently, we investigated the expression of these RBPs in the RNA‐seq files of SAHA and TSA treated cells. We found that SAHA had no effect on all these RBPs, while TSA had a weak effect on 4 RBPs (ACO1, KHDRBS3, MBNL1, and EIF4B) (Figure S6B, Supporting Information). As we did not get consistent and significant results in both SAHA and TSA treatment cells, so we suggested HDACi may not affect FSP1 by RBPs. In further detection, we demonstrated that HDACi reduced the mRNA expression of FSP1 and accelerated the mRNA degradation of FSP1 (**Figure** **3**A,B; Figure S6C, Supporting Information). As it is known, RNA methylation significantly influences RNA stability. Consequently, we conducted additional analysis to identify the primary types of RNA modifications regulated by HDACi using Liquid chromatography‐mass spectrometry (LC/MS). Interestingly, HDACi significantly repressed the level of m^6^A RNA methylation but showed no effect on m^1^A, m^5^C, and m^7^G RNA methylation (Figure 3C). We also confirmed that HDACi reduced m^6^A RNA methylation of total mRNA through Dot Blot analysis (Figure 3D). Subsequently, we observed substantial m^6^A enrichment in FSP1 mRNA using meRIP‐qPCR. Furthermore, treatment with HDACi notably diminished m^6^A enrichment in FSP1 mRNA (Figure 3E; Figure S6D, Supporting Information). To explore the mechanism behind the repression of m^6^A enrichment by HDACi, we delved into the transcriptional profiles of HDACi‐treated HCT116. Our investigation revealed that both SAHA and TSA heightened the expression of two primary m^6^A writers, FTO and ALKBH5, a finding further validated through RT‐qPCR and Western Blotting (Figure 3F,G; Figure S6E, Supporting Information). Consistent with the in vitro findings, SAHA also enhanced the expression of both FTO and ALKBH5 in AOM/DSS‐induced CRC (Figure S6F,G, Supporting Information). Following this, we individually overexpressed FTO and ALKBH5 in CRC cell lines (Figure 3H). The overexpression of both FTO and ALKBH5 led to a reduction in m^6^A levels and mRNA stability of FSP1, consequently resulting in decreased FSP1 expression (Figure 3I–L; Figure S6H, Supporting Information). Consistent with the reduction in FSP1 levels, the overexpression of FTO and ALKBH5 also amplified ferroptosis induced by various FINs (RSL3, erastin, and FINO_2_) (Figure 3M,N). The overexpression of FTO and ALKBH5 did not alter the reduced cell viability induced by lethal dose HDACi (SAHA, TSA, and SB) (Figure S6I, Supporting Information). In light of HDACi's mechanism involving the promotion of histone acetylation, we proposed that HDACi regulates FTO and ALKBH5 through histone acetylation. As anticipated, H3K9ac, H3K14ac, H3K27ac, H3K56ac, and H4K16ac showed enrichment in the promoter regions of FTO and ALKBH5. Furthermore, treatment with SAHA only resulted in increased enrichments of H3K27ac (Figure 3O). Considering these findings collectively, we proposed that HDACi enhances the H3K27ac of FTO and ALKBH5, consequently increasing their expression. Therefore, the overexpression of FTO and ALKBH5 decreases the m^6^A modification of FSP1 mRNA, thereby accelerating the degradation of FSP1 mRNA.

### 2.4 IGF2BP1 Reads m^6^A Modification on FSP1 mRNA

**Figure 4:**
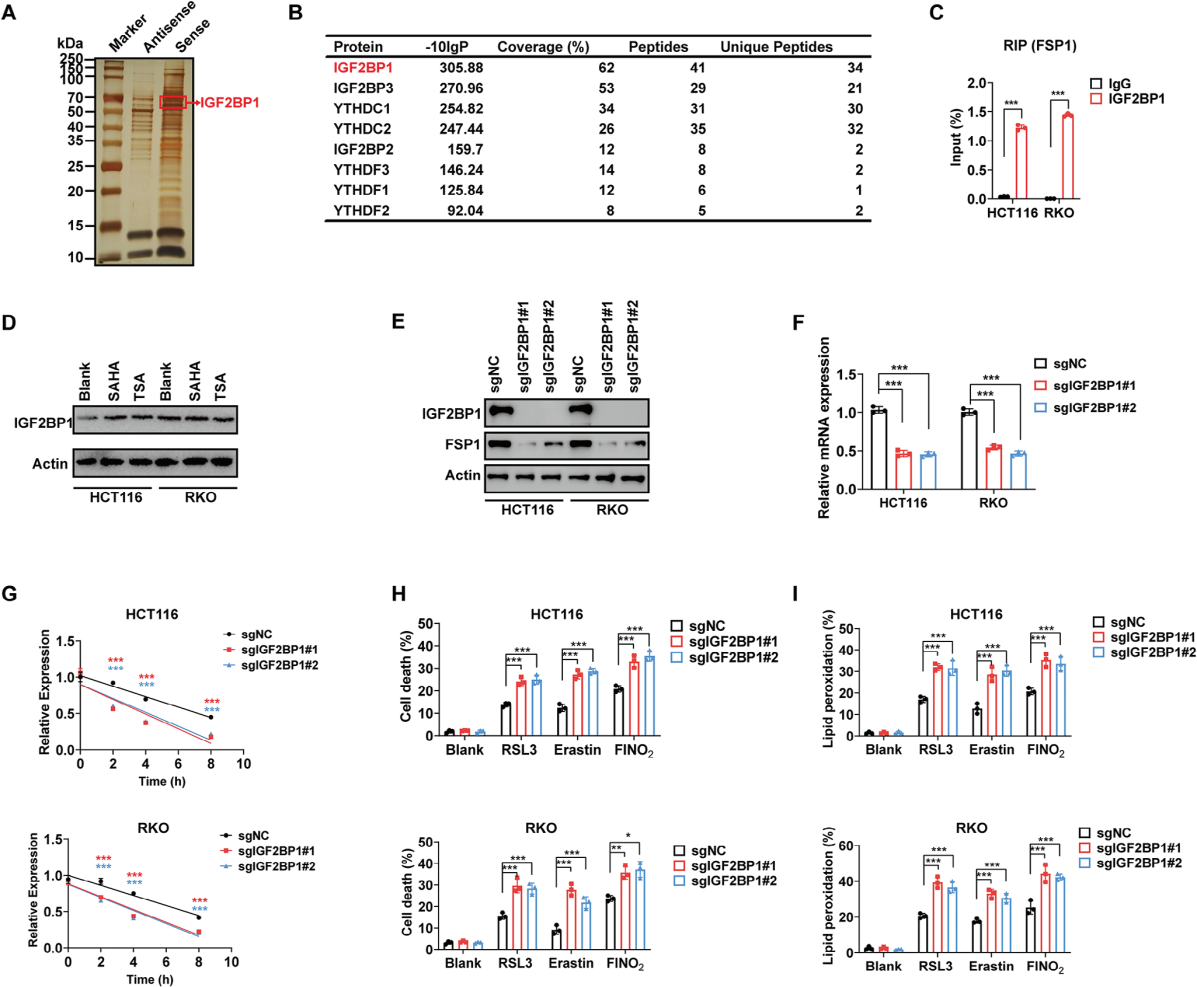
IGF2BP1 reads m^6^A modification of FSP1 mRNA. A) Silver stain of immunoprecipitated products of FSP1 sense and antisense probes. B) Potential FSP1 m^6^A readers revealed by RNA pull‐down MS. C) Enrichment of FSP1 mRNA in IGF2BP1 immunoprecipitated products revealed by RIP‐qPCR. D) Protein expression of IGF2BP1 in CRC cells treated with SAHA and TSA. E–G) Protein expression (E), mRNA expression (F), and mRNA stability (G) of FSP1 in IGF2BP1‐depleted CRC cells. H,I) Cell death (H) and lipid peroxidation of IGF2BP1‐depleted CRC cells treated with different FINs (RSL3: 5 µM, erastin: 40 µM, FINO_2_: 20 µM). (^*^ *p* < 0.05, ^**^ *p* < 0.01, ^***^ *p* < 0.001).

Using RNA pull‐down MS assay, we observed substantial enrichment of m^6^A readers in the FSP1 mRNA probe, with IGF2BP1 displaying the most significant binding (**Figure** **4**A,B). Additionally, our confirmation of the interaction between IGF2BP1 protein and FSP1 mRNA through RIP assay suggests that IGF2BP1 serves as the m^6^A reader for FSP1 (Figure 4C). Meanwhile, we observed that HDACi did not impact the expression of IGF2BP1 (Figure 4D). Upon depleting the expression of IGF2BP1 in CRC cells, we confirmed that its depletion repressed the mRNA stability, mRNA expression, and protein expression of FSP1 (Figure 4E–G). In line with these findings, the depletion of IGF2BP1 also sensitized CRC cells to various FINs (RSL3, erastin, and FINO_2_) (Figure 4H,I). Taken together, these data confirm that HDACi diminishes m^6^A RNA methylation and accelerates the degradation of FSP1 mRNA by upregulating FTO and ALKBH5. Our results indicate that IGF2P1 acts as the reader for FSP1.

### 2.5 HDAC1 Serves as the Main Target for HDACi‐Regulated Ferroptosis

**Figure 5:**
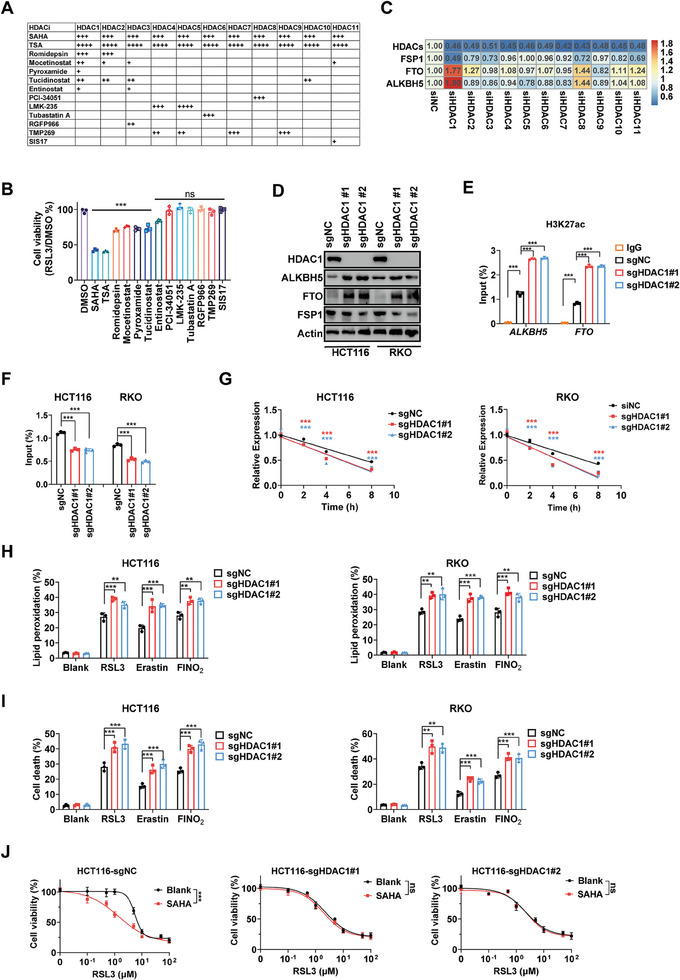
HDAC1 serves as the main target for HDACi‐regulated ferroptosis. A) Target list of different HDACi. “+/√” represents IC50. ++++: <10 nm +++: 10–50 nm, ++: 50–200 nm, +: >200 nm, √: no IC50 data. (https://www.selleck.cn/). B) Relative cell viability of HCT116 cells treated with different HDACi (LMK‐235: 0.1 µm; romidepsin: 0.05 µm; mocetinostat: 0.5 µm; TSA: 10 µm; TMP269: 0.5 µm; PCI‐34051: 50 µm; pyroxamide: 1 µm; RGFP966: 10 µm; SIS17: 10 µm; tucidinostat: 1 µm; entinostat: 1 µm) and RSL3 for 24 h. C) mRNA expression of HDACs, FSP1, FTO, and ALKBH5 in HCT116 cells transfected with different HDAC siRNAs. D) Expression of FSP1, FTO, and ALKBH5 in HDAC1‐depleted CRC cells. E–I) H3K27ac in the promoters of *FTO* and *ALKBH5* (E), m^6^A modification in FSP1 mRNA (F), mRNA stability of FSP1 (G), lipid peroxidation (H, detected at 12 h), and cell death (I, detected at 24 h) induced by different FINs (RSL3: 5 µm, erastin: 40 µm, FINO_2_: 20 µm) in HDAC1‐depleted CRC cells. J) The response curve of wild‐type and HDAC1‐depleted HCT116 cells to multiple doses of RSL3 (0–100 µm, 24 h) with or without combining sublethal concentration of SAHA (1 µm). (^**^ *p* < 0.01, ^***^ *p* < 0.001).

Given that SAHA and TSA are pan‐HDACi that target all members of the HDAC family, we proceeded to investigate the specific roles of individual HDACs in regulating ferroptosis. Following a screening of an HDACi library targeting specific HDACs, we observed that sublethal doses of pyroxamide, romidespin, mocetinostat, tucidinostat, and entinostat sensitized RSL3‐induced ferroptosis. Notably, these compounds shared HDAC1 as a cross target (**Figure** **5**A,B), and detailed dose‐response curves of these HDACi are provided in Figure S7 (Supporting Information). We employed a siRNA library to individually knock down the expression of each HDAC. Upon knockdown of HDAC1, we observed a reduction in the mRNA expression of FSP1, along with increased expression of ALKBH5 and FTO (Figure 5C). Subsequently, we confirmed that the depletion of HDAC1 led to reduced protein expression of FSP1 while promoting the expression of ALKBH5 and FTO (Figure 5D). We also found that the depletion of HDAC1 promoted the enrichment of H3K27ac in the promoters of ALKBH5 and FTO, while simultaneously decreasing m^6^A modification and mRNA stability of FSP1 (Figure 5E–G). Finally, our findings also supported the idea that depletion of HDAC1 sensitized cells to ferroptosis induced by different FINs (Figure 5H,I). Meanwhile, SAHA sensitized ferroptosis in wild‐type HCT116 cells; however, this sensitization was not observed in HDAC1‐depleted HCT116 cells (Figure 5J). Collectively, these results indicate that HDAC1 serves as the primary target for HDACi‐regulated ferroptosis.

### 2.6 Lactylation of HDAC1^K412^ is Essential for Ferroptosis Regulation

**Figure 6:**
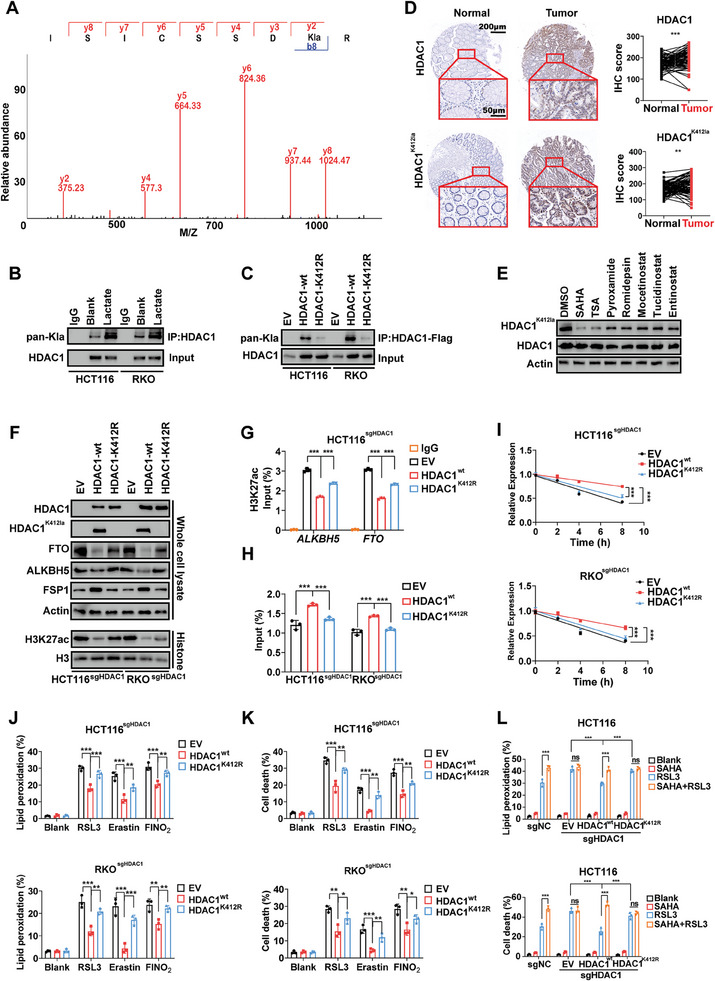
Lactylation of HDAC1^K412^ is essential for ferroptosis regulation. A) Lactylation of HDAC1^K412^ in CRC tissues. B) Lactate (10 mm, 24 h) enhanced HDAC1^K412^ lactylation in CRC cells. C) Mutation of HDAC1^K412^ abolished HDAC1 lactylation. D) IHC staining of HDAC1 and HDAC1^K412^ lactylation (HDAC1^K412la^) in CRC and paired normal tissues (*n* = 79). E) Expression of HDAC1^K412la^ and HDAC1 in CRC cells treated with SAHA and RSL3. F) Expression of HDAC1^K412la^, HDAC1, FTO, ALKBH5, FSP1, and H3K27ac in HDAC1‐depleted CRC cells transfected with HDAC1 ^wt^ and HDAC^K412R^. G–K) H3K27ac in the promoters of *FTO* and *ALKBH5* (G), m^6^A modification in FSP1 mRNA (H), mRNA stability of FSP1 (I), lipid peroxidation (J) and cell death (K) induced by FINs in HDAC1‐depleted CRC cells transfected with HDAC1 ^wt^ and HDAC^K412R^. L) Lipid peroxidation and cell death induced by FINs and SAHA in HDAC1‐depleted HCT116 cells transfected with HDAC1 ^wt^ and HDAC^K412R^. (*ns*: no significance, ^*^ *p* < 0.05, ^**^ *p* < 0.01, ^***^ *p* < 0.001).

Lactylation involves the accumulation of lactate during cellular metabolism, altering lysine residues on histones, and plays a pivotal role in cancer development.^[^ ^18a^ ^]^ HDAC1 has been identified as an enzyme that removes acetylation from histones. Our previous research has shown that lactate promotes resistance to ferroptosis within the hypoxic tumor microenvironment.^[^ ^24^ ^]^ Therefore, we propose that lactylation plays a key role in HDAC1‐regulated ferroptosis. Intriguingly, through LC/MS analysis of CRC tissues, we unexpectedly discovered significant lactylation on HDAC1K412 (**Figure** **6**A). Subsequent IP assays in CRC cells further confirmed that the presence of lactylation on HDAC1 was augmented by the exogenous addition of lactate (10 mm, 24 h) (Figure 6B). Simultaneously, the mutation of HDAC1K412 (HDAC1K412R) notably abolished its lactylation (Figure 6C). We then developed a specific rabbit polyclonal antibody targeting HDAC1K412 lactylation to assess its presence in CRC tissues. Remarkably, both HDAC1 and HDAC1^K412^ lactylation were significantly overexpressed in CRC tissues in comparison to paired normal bowel tissues (Figure 6D). Both SAHA and TSA significantly reduced HDAC1K412 lactylation, but not that of HDAC1 in CRC cells (Figure 6E). In line with the results in vitro, SAHA also significantly repressed HDAC1^K412^ lactylation in the AOM/DSS‐induced CRC mouse model (Figure S8, Supporting Information). Other HDAC1 inhibitors (pyroxamide, etc.) showed moderate repression on HDAC1^K412^ lactylation, but not as significant as TSA and SAHA, which is consistent with the trend of sensitizing effect to ferroptosis. Subsequently, we restored wild‐type HDAC1 (HDAC1 ^wt^) and HDAC1^K412^ mutation (HDAC1^K412R^) in HDAC1‐depleted CRC cells to learn the role of HDAC1^K412^ lactylation in ferroptosis regulation. Compared to HDAC1 ^wt^, HDAC1^K412R^ showed a significantly impaired effect on promoting FSP1 and repressing FTO and ALKBH5. We observed that HDAC1^K412R^ also exhibited weaker repression of H3K27ac in comparison to HDAC1 ^wt^ (Figure 6F). Consistent with the regulation of FSP1, FTO, and ALKBH5, CRC cells expressing HDAC1^K412R^ displayed higher enrichment of H3K27ac in the promoters of FTO and ALKBH5, along with lower m^6^A modification and mRNA stability of FSP1 mRNA (Figure 6G–I). Additionally, CRC cells with HDAC1^K412R^ were more sensitive to FINs compared to CRC cells with HDAC1 ^wt^ (Figure 6J,K). Notably, SAHA failed to sensitize RSL3‐induced ferroptosis in CRC cells with HDAC1^K412R^ (Figure 6L). Overall, our findings confirmed that HDAC1^K412^ lactylation is crucial in regulating the m^6^A‐FSP1‐ferroptosis axis in CRC.

## 3 Discussion

The potency of traditional cytotoxic drugs resides in their capacity to inhibit tumor growth by initiating apoptosis. Nonetheless, the development of resistance in tumor cells toward therapy remains an insurmountable challenge.^[^ ^25^ ^]^ The exploration of non‐apoptotic programmed cell death introduces an additional therapeutic strategy for impeding tumor growth. Notably, the efficacy of ferroptosis as a therapeutic strategy for tumors has been well‐established. However, the resistance of CRC, a highly malignant form of cancer, to ferroptosis poses a significant obstacle to the implementation of ferroptosis therapy.^[^ ^4^, ^26^ ^]^ There are currently no clinically approved drugs for ferroptosis. The existing specific inhibitor of FSP1, iFSP1, is not suitable for use in vivo, because it has off‐target effects at high concentrations, and cannot target FSP1, promoting cell ferroptosis, which limits its clinical transformation.^[^ ^27^ ^]^ HDAC removes the acetyl group from histone lysine residue, alters its charge, tightens chromatin structure, and inhibits transcriptional expression of genes.^[^ ^28^ ^]^ HDACi can induce apoptosis, autophagy, necrosis, and ROS generation in cancer cells. They can also impede cell cycle progression and inhibit cancer cell proliferation, thereby facilitating effective cancer treatment.^[^ ^29^ ^]^ Based on previous research and through our drug screening efforts, we demonstrated that HDACi sensitized CRC to ferroptosis, thus promoting the application of ferroptosis therapy in CRC. While HDACi were historically presumed to curb tumor cell proliferation by inducing cell cycle arrest, differentiation, and/or apoptosis, our research delineated their promising regulatory effect on ferroptosis. Traditionally employed primarily as first‐line therapy in hematological tumors, HDACi has transitioned toward secondary or being a part of combination therapy in solid tumors.^[^ ^22^ ^]^ Bolstered by their potential to sensitize cells to ferroptosis, the amalgamation of HDACi and ferroptosis inducers stands to broaden the applicability of HDACi across various solid tumors.

The xCT‐GSH‐GPX4 and FSP1‐CoQH_2_ systems represent the primary defense mechanisms against ferroptosis.^[^ ^2^, ^30^ ^]^ The extracellular uptake of cysteine through the xCT transporter, coupled with intracellular glutamate release, is the main regulatory pathway for cell ferroptosis. In the cytoplasm, cysteine undergoes conversion into cysteine, serving as the main precursor for glutathione (GSH) synthesis. GSH acts as an electron donor and enzymatically reduces toxic lipid peroxides to non‐toxic alcohols through GPX4‐mediated hydrolysis. However, after the inactivation of GPX4, certain tumor cells exhibit resistance to ferroptosis, indicating the presence of alternative mechanisms for defending against this form of cell death.^[^ ^31^ ^]^ Conversely, FSP1 functions as an oxidoreductase by reducing CoQ to CoQH_2_ at the cellular membrane.^[^ ^32^ ^]^ The lipophilic antioxidant CoQH_2_ effectively scavenges free radicals and inhibits lipid peroxidation, thereby demonstrating its potential as a therapeutic agent.^[^ ^21^ ^]^ Our research findings further confirm the upregulation of FSP1 in CRC and its crucial role as a primary defense mechanism against ferroptosis. The inhibition of these two tumor defense systems is anticipated to emerge as a pivotal strategy for enhancing the therapeutic efficacy of ferroptosis.

As noted, while there exists a plethora of drugs targeting the SLC7A11‐GSH‐GPX4 system to induce ferroptosis, there is a noticeable scarcity of drugs that specifically target the FSP1 system. iFSP1, serving as a selective and GSH‐independent inhibitor of FSP1, has largely undergone in vitro studies and still awaits clinical translation.^[^ ^21^ ^]^ However, our study reveals that HDACi, particularly SAHA, serve as potent inhibitors of FSP1.

Our experimental data suggest that the combination of HDACi and ferroptosis inducers synergically inhibit CRC growth in vivo and in vitro. We have proved that HDACi drives response to ferroptosis inducer by repressing FSP1. Given the widespread usage of HDACi in clinical settings, their potential as promising agents for inducing ferroptosis comes to the forefront in this context. Considering that HDACi has some side effects in clinical application, in order to enhance activity and efficacy while minimizing toxicity associated with off‐target effects, the development of HDAC inhibitors with potent activity and high selectivity has emerged as a crucial area of research and development. The development of dual (multitarget) inhibitors is also a promising research direction for HDAC inhibitors. If these two or more targets exhibit coordinated effects, improved efficacy can be anticipated. Additionally, drug combinations represent a crucial strategy for broadening the application of HDAC inhibitors in various indications, including solid tumors.

Through screening siRNA libraries and HDACi libraries, we confirmed that HDAC1 plays a pivotal role as the primary regulator of ferroptosis sensitization in response to HDACi treatment. Serving as a histone deacetylase, HDAC1 primarily reduces histone acetylation and inhibits gene transcription. Yet, the remarkable observation of abnormal phenomena in HDAC1 knockout/HDACi‐induced decrease in FSP1 transcriptional expression suggests that HDAC1 does not directly target histones enriched in the FSP1 gene. Further investigation revealed that HDAC1 knockout/HDACi effectively prompted the degradation of FSP1 mRNA and reduced its mRNA stability. The stability of mRNA is chiefly regulated by RNA modifications, comprising over 100 identified types. Among these modifications, RNA methylation, notably m^6^A (N6 methyladenine, 6‐methyladenine) and uridylation (U‐tail), stands out as the most prevalent in eukaryotes.^[^ ^33^ ^]^ Interestingly, our findings indicate a substantial presence of m^6^A modifications on FSP1 mRNA, which is regulated by HDAC1/HDACi in terms of its modification level. Notably, the members of the m^6^A eraser complex are represented by FTO and ALKBH5, both of which belong to the Fe2+/α‐ketoglutarate‐dependent dioxygenase family.^[^ ^34^ ^]^ They possess the ability to recognize the methylation of adenine and cytosine in single‐stranded DNA and RNA molecules. In thyroid cancer, studies have reported that FTO regulates ferroptosis responsiveness by inhibiting m^6^A modification of SLC7A11 mRNA, while ALKBH5 regulates ferroptosis in thyroid cancer through the classical antioxidant stress pathway Nrf2/HO‐1.^[^ ^35^ ^]^ However, documentation regarding the regulation of ferroptosis by these two key m^6^A erasers in other tumors remains limited. Our observations indicated that the knockout of HDAC1 or treatment with HDACi notably enhanced the expression of two m^6^A erasers, namely ALKBH5 and FTO. This led to the downregulation of m^6^A modification on FSP1 mRNA, facilitating its degradation and ultimately promoting cellular response to ferroptosis. Chromatin analysis of HDAC1 demonstrated its predominant targeting of the acetylation of K27 on histone H3 (H3K27ac) on both FTO and ALKBH5. Finally, the functional roles of FTO and ALKBH5 as m^6^A erasers, along with IGF2BP1 as an m^6^A reader for FSP1, were ultimately confirmed.

In our previous studies, we have established the significance of the acidic microenvironment in solid tumors such as CRC, where hypoxia‐induced lactate production plays a crucial role in conferring resistance to ferroptosis.^[^ ^24^ ^]^ Local lactate accumulation will lead to increased levels of lactylation, a novel lactate‐derived post‐translational modification of histones.^[^ ^36^ ^]^ However, the specific molecular mechanism by which lactate regulates ferroptosis and its relationship with lactylation modification remains unclear. Our research unveils a new lactylation site (K412) in HDAC1, shedding light on its role in maintaining the function of HDAC1 in regulating ferroptosis through its influence on histone deacetylase activity. Remarkably, while previous reports have primarily focused on lactylation occurring on histones, our study reveals the presence of lactylation modification on HDACs, demonstrating an extension of its functional reach.^[^ ^18^ ^]^

In accordance with in vitro research, preclinical studies utilizing AOM/DSS‐induced CRC models demonstrated that SAHA sensitized tumor cells to FIN IKE. The combination of SAHA with IKE exhibited potential advantages in terms of safety compared to commonly used CRC chemotherapy agents (5‐FU plus OXA). In clinical practice, the combination of 5‐FU plus OXA commonly leads to gastrointestinal adverse reactions (including diarrhea, nausea, vomiting, and mucositis), hematological adverse reactions (such as neutropenia and thrombocytopenia), as well as neurological reactions (including acute, dose cumulative peripheral sensory neuropathy).^[^ ^37^ ^]^ In our in vivo study, no adverse reactions were observed in the SAHA+IKE treatment group, underscoring its potential to provide a favorable safety profile.

**Figure 7:**
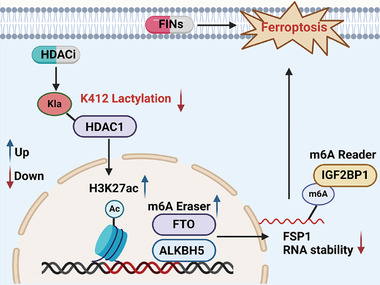
Schematic working model for HDAC1 lactylation‐driven ferroptosis resistance in CRC.HDAC inhibitors (SAHA and TSA) induce a reduction in lactylation of HDAC1^K412^, leading to the inhibition of HDAC1 expression and an increase in H3K27 histone acetylation modification. The enhanced histone acetylation modification of H3K27 promotes transcriptional activation of m6A Eraser enzymes (FTO and ALKBH5), resulting in increased expression. Consequently, this leads to a decrease in the m6A modification of FSP1, reduced stability of FSP1, and ultimately decreased levels of FSP1, thereby enhancing sensitivity to ferroptosis.

In conclusion, our study unveiled that HDACi specifically targeted HDAC1 and promoted the H3K27ac modification of FTO and ALKBH5. The resulting activation of FTO and ALKBH5 led to a reduction in m^6^A modification on FSP1 mRNA, culminating in its degradation and ultimately sensitizing CRC to ferroptosis. We revealed the intricate interplay between histone modifications and RNA methylation modifications. The diminishment of HDAC1^K412^ lactylation by HDACi is crucial for regulating ferroptosis (**Figure** **7**). Altogether, our study may significantly advance our understanding of the critical mechanisms related to HDACi‐induced decrease in HDAC1 lactylation. The combination of HDACi and FIN treatment represents an innovative and potent therapeutic strategy for managing CRC, thereby addressing a pressing need in oncology research and clinical practice.

## 4 Experimental Section

### Patients and Specimens

The CRC specimens and paired non‐tumor bowel tissues were collected from June 2021 to March 2023 at the Wuxi People's Hospital Affiliated to Nanjing Medical University. Patients meeting the following criteria were excluded from participation: those who had undergone adjuvant chemotherapy or radiotherapy before surgery, and those with additional cancer diagnoses. All patients were categorized based on the 8th edition of the TNM staging system. Postoperative adjuvant therapies were administered according to standardized schedules and doses. Written informed consent was obtained from all participating patients. The procedures were performed in accordance with the Declaration of Helsinki. This study received approval from the Ethical Committee of Nanjing Medical University (NJMU‐ER‐2023‐315). Detailed clinical features of CRC patients are provided in Table S1 (Supporting Information).

### Cell Culture and Reagents

The human cancer cell lines HCT116, HepG2, RKO, MDA‐MB‐231, Hela, U2OS, HT1080, SW1990, Caki1, PC3, TPC1, and A549 were obtained from the Cell Bank of the University of Colorado Cancer Center. The cells were cultured in DMEM or RPMI 1640 (Gibco, CA, USA) medium supplemented with 10% FBS (Invitrogen, CA, USA) at 37 °C in a cell incubator containing 5% CO_2_. 5‐Fluorouracil (5‐FU, HY‐90006), oxaliplatin (OXA, HY‐17371), imidazole ketone erastin (IKE, HY‐114481), liproxstatin‐1 (Lip1, HY‐12726), RSL3 (HY‐100218A), erastin (HY‐15763), doxorubicin (HY‐15142A), irinotecan (HY‐16562), capecitabine (HY‐B0016), etoposide (HY‐13629), vemurafenib (HY‐12057), SAHA (HY‐10221), binimetinib (HY‐15202), cetuximab (HY‐P9905), ML210 (HY‐100003), FINO_2_ (HY‐129457), DFO (HY‐B0988), necrostatin‐1 (HY‐15760), Z‐VAD‐FMK (HY‐16658B), pyroxamide (HY‐13216), and TSA (HY‐15144) were purchased from MedChemExpress (Monmouth Junction, NJ, USA). Sodium butyrate (SB, S1999), LMK‐235 (S7569), romidepsin (S3020), mocetinostat (S1122), tubastatin A (S8049), TMP269 (S7324), PCI‐34051 (S2012), RGFP966 (S7229), SIS17 (S6687), tucidinostat (S8567), and entinostat (S1053) were purchased from Selleck Chemicals (Houston, TX, USA). TNF‐α (RP00001) was purchased from ABclonal Technology (Wuhan, China).

### Measurement of Cell Viability, Cell Death, and Lipid Peroxidation

For the assessment of cell viability, 10^4^ cells per well were seeded overnight in 100 µl of medium within 96‐well plates. Subsequently, the CRC cell lines (HCT116, RKO, SW620, HT29) were exposed to various ferroptosis inducers (FINs)/strategies including RSL3, erastin, and cystine starvation for a duration of 24 h. Finally, cell viability was determined by adding 10 µl of CCK‐8 reagent (Dojindo Molecular Technologies, Kumamoto, Japan, CK04) per well followed by incubation for 2 h and measurement of absorbance at a wavelength of 450 nm (OD 450). In the assays of combination therapy (HDACi with FINs, targeted therapy or chemotherapy reagents with FINs), sublethal doses (lethal concentration/LC10‐30) of HDACi, targeted therapy, and chemotherapy reagents were adopted according to the dose‐response curves. Multiple doses of RSL3 and erastin were applied. For the measurement of lipid peroxidation and cell death, 10^6^ cells were seeded in 2 mL of medium in 6‐well plates and incubated overnight. Subsequently, the cells were treated with specified concentrations of RSL3, erastin, FINO_2_, SB, TSA, and SAHA for a duration of 24 h. To detect lipid peroxidation, the cells were incubated with 2 mm C11 BODIPY 581/591 (Invitrogen, Waltham, MA, USA, D3861). For the detection of cell death, cells were incubated with propidium iodide (PI) (Invitrogen, R37169) at a concentration of 1 mg mL^−1^ in PBS. Following two washes with PBS, fluorescence‐activated cell sorting (FACS) analysis was performed on the collected cells using the FL1 channel for C11 BODIPY and the FL2 channel for PI.

### Intestinal Organoid Culture and Activity Detection

The mouse intestinal crypts were isolated from 8 to 12‐week‐old C57BL/6 mice (Jackson Laboratory) following euthanasia by CO₂ asphyxiation. The crypts were extracted using cold PBS containing 10 mm EDTA, followed by mechanical dissociation through vigorous shaking and filtration through a 70‐µm cell strainer (Corning, Cat. #431751). Subsequently, the isolated crypts were embedded in 30 µL of growth‐factor reduced Matrigel (Corning, Cat. #356231) and overlaid with a complete organoid culture medium. This medium consisted of Advanced DMEM/F‐12 (Thermo Fisher Scientific, Cat. #12634010), EGF at a concentration of 50 ng mL^−1^ (PeproTech, Cat. #315‐09), Noggin at a concentration of 100 ng mL^−1^ (PeproTech, Cat. #250‐38), and R‐spondin 1 at a concentration of 500 ng mL^−1^ (R&D Systems, Cat. #3474‐RS). The cultures were maintained at a temperature of 37 °C with an atmosphere containing 5% CO₂, and the medium was refreshed every 2–3 days. The CellTiter‐Glo Luminescent Assay (Promega, Cat. #G7570) was employed to evaluate cell viability by measuring ATP levels as an indicator of metabolic activity. Organoids were cultured in 96‐well plates, and after treatment, 100 µL of the reagent was added per well. Following a 10 min incubation at room temperature, luminescence readings were obtained using a BioTek Synergy H1 microplate reader.

### Animal Studies

The detailed experimental procedures were approved by the Institutional Animal Care and Use Committee of Nanjing Medical University (IACUC‐2410014). 36 six‐week old male BALB/c nude mice were obtained from Beijing Vital River Laboratory Animal Technology and raised in the Animal Center of Nanjing Medical University. A total of 10^6^ HCT116 cells suspended in 100 µL of PBS were subcutaneously injected into the axilla of each nude mouse. After 10 days, the mice were randomly and equally divided into the designated groups (n = 6 per group), treated with 10% DMSO solvent (10% DMSO, 40% PEG300, 5% Tween 80, 45% saline) control, IKE (30 mg kg^−1^, i.p., per day), SAHA (50 mg kg^−1^, i.p., per day), 5‐fluorouracil (5‐FU, 5 mg kg^−1^, i.p., per 3 days), oxaliplatin (OXA, 5 mg kg^−1^, i.p., per 3 days), liproxstatin‐1 (Lip1, 10 mg kg^−1^, i.p., per day). OXA was dissolved in saline. IKE, SAHA, 5‐FU, and Lip1 were dissolved in 10% DMSO solvent. The long (L) and short (S) diameters of tumors were measured every four days to calculate tumor volume using the formula L^*^S^2^/2. Growth curves for subcutaneous tumors were plotted based on tumor volume measurements. At the end of the administration period, all mice were euthanized and their subcutaneous tumors were completely excised. The tumors were weighed and embedded in paraffin for sectioning purposes. Mice reaching a xenograft diameter of ≥20 mm, xenograft volume ≥2000 mm^3^, or experiencing a body weight loss ≥20% were euthanized using an overdose of sodium pentobarbital diluted in saline (100 mg kg^−1^, diluted in saline). For the azoxymethane/dextran sulfate sodium (AOM/DSS) model of CRC, male C57BL/6 N mice at six weeks of age were obtained from Beijing Vital River Laboratory Animal Technology Co., Ltd. Following 1 week of adaptive feeding, all mice received a single intraperitoneal injection of 10 mg kg^−1^ AOM (once, i.p. injection). Subsequently, they were provided with drinking water containing 2% DSS (MP Biomedicals, Irvine, CA, USA) for 5 days, followed by regular drinking water for 16 days. This cycle of DSS/regular drinking water was repeated 3 times, and then regular drinking water was given for an additional 14 days. During the last 30 days (42‐72 days) of this experimental procedure, the mice were treated with the same strategies described in the xenografts model above. All mice were sacrificed at day 77 or if they lost more than 20% of body weight using an overdose of sodium pentobarbital (100 mg kg^−1^, diluted in saline).

### Quantification and Statistical Analysis

All experiments were independently conducted at least 3 times. Statistical analysis of all experimental data was performed using SPSS software (version 22.0, IBM Corp., Armonk, NY, USA). GraphPad Prism (version 8, GraphPad Software, La Jolla, CA, USA) was utilized for analyzing the statistical results. All data were presented as mean ± standard deviation (mean±SD). Student's t‐test was employed for comparing data between two groups. One‐way ANOVA followed by Fisher's least significant difference (LSD) Test was used for comparisons among multiple groups. Differences with a significance level of *P* < 0.05 were considered statistically significant.

### Ethics Approval and Consent to Participate

All procedures involving human participants were performed in accordance with the Ethics Committee of Nanjing Medical University and with the 1964 Declaration of Helsinki and its later amendments or comparable ethical standards. All patients provided written informed consent. The study protocol was approved by the Ethics Committee of Nanjing Medical University.

### Availability of Data and Materials

RNA‐seq profiles were deposited in the GEO database (GSE263463). These data will be publicly available once the manuscript has been accepted. Other datasets used and analyzed during the current study were available from the corresponding author upon reasonable request.

### Patient Consent for Publication

Written informed consent for publication was obtained from all the participants.

## Conflict of Interest

The authors declare no conflict of interest.

## Author Contributions

Z.Y., W.S., Q.Z., and L.N. contributed equally to this work. Z.Y. and W.S. conducted the experiments and wrote the manuscript. Q.Z. and L.N. contributed to the statistical analysis of the data. B.F. and Y.Z. performed the animal studies. F.H. and J.H. contributed to resources and data curation, Q.Z. and X.Z. contributed to validation, L.M. and J.Z. contributed to project administration, Y.W. and W.X. contributed to funding acquisition, J.X. and Z.H. contributed to writing review, and Q.Z. and B.Y. contributed to the design and supervision of the study. All authors read and approved the final version of the manuscript.

## Supporting information



Supporting Information

Supplemental Table 6

## Data Availability

The data that support the findings of this study are available from the corresponding author upon reasonable request.
