# Supplementary material for: Lactylation of HDAC1 Confers Resistance to Ferroptosis in Colorectal Cancer
Source: Adv Sci (Weinh). 2025 Jan 31;12(12):2408845. doi: 10.1002/advs.202408845 (PMC11947995; doi:10.1002/advs.202408845)
Supplement: Supplementary file 1 — Supporting Information [file ADVS-12-2408845-s002.docx]

Supplementary file for

**Lactylation of HDAC1 confers resistance to ferroptosis in colorectal cancer**

Zhou Yang^1, 2, #^, Wei Su^2, 3, #^, Qinglin Zhang^4, #^, Lili Niu^5, #^, Baijie Feng^6^, Yu Zhang^1, 2^, Feng Huang^7^, Jiaxin He^7^, Qinyao Zhou^7^, Xin Zhou^7^, Longjun Ma^8^, Jingwan Zhou^7^, Yuanrong Wang^7^, Wenjing Xiong^7^, Jun Xiang^1, 2, *^, Zhilin Hu^9, *^, Qiang Zhan^4, *^, Bing Yao^7, 10, *^

^1^Department of Head and Neck Surgery, Fudan University Shanghai Cancer Center, Shanghai, China

^2^Department of Oncology, Shanghai Medical College, Fudan University, Shanghai, China

^3^Department of Medical Oncology, Fudan University Shanghai Cancer Center, Shanghai, China

^4^Departments of Gastroenterology, Wuxi People’s Hospital Affiliated to Nanjing Medical University, Nanjing Medical University, Nanjing, Jiangsu Province, China

^5^Department of Integrative Medicine, Shanghai Pulmonary Hospital, Tongji University Medical School Cancer Institute, Tongji University, Shanghai, China

^6^Department of Medical Oncology, Shanghai Pudong Hospital, Fudan University Pudong Medical Center, Shanghai, China

^7^National Experimental Teaching Center of Basic Medical Science, School of Basic Medical Sciences, Nanjing Medical University, Nanjing, China

^8^Department of Epidemiology, School of Public Health, Nanjing Medical University, Nanjing, China

^9^Department of Immunology, Key Laboratory of Immune Microenvironment and Disease, The School of Basic Medicine, Nanjing Medical University, Nanjing, China

^10^Department of Medical Genetics, School of Basic Medical Sciences, Department of General Surgery, The Affiliated Taizhou People's Hospital of Nanjing Medical University, Taizhou School of Clinical Medicine, Nanjing Medical University

^#^These authors contributed equally

^*^Co-corresponding authors:

Bing Yao, Department of Medical Genetics, School of Basic Medical Sciences, Nanjing Medical University, Nanjing, China; Email: [byao@njmu.edu.cn](mailto:byao@njmu.edu.cn)

Qiang Zhan, Departments of Gastroenterology, Wuxi People’s Hospital Affiliated to Nanjing Medical University, Nanjing Medical University, Nanjing, Jiangsu Province, China; Email: ryzhanqiang@njmu.edu.cn

Zhilin Hu, Department of Immunology, Key Laboratory of Immune Microenvironment and Disease, The School of Basic Medicine, Nanjing Medical University, Nanjing, China; Email: hzl1126514@njmu.edu.cn

Jun Xiang, Department of Head and Neck Surgery, Fudan University Shanghai Cancer Center, Shanghai 200032, China; Email: xiangjun@shca.org.cn

**File list**

Table S1-S5

Figure S1-S7

**Expanded Material and methods**

***Measurement of cell viability, cell death and lipid peroxidation***

For the assessment of cell viability, 10^4^ cells were seeded overnight in 100 μl of medium within 96-well plates. Subsequently, the CRC cell line (HCT116, RKO, SW620, HT29) was exposed to various ferroptosis inducers (FINs)/strategies including RSL3, Erastin, and Cystine starvation for a duration of 24 h. Finally, cell viability was determined by adding 10 µl of CCK-8 reagent (Dojindo Molecular Technologies, Kumamoto, Japan, CK04) per well followed by incubation for 2 h and measurement of absorbance at a wavelength of 450 nm (OD 450). In the assays of combination therapy (HDACi with FINs, or targeted therapy/chemotherapy reagents with FINs), sublethal doses (LC10-30) of HDACi, targeted therapy and chemotherapy reagents were adopted in according with dose-response curves. Multiple doses of RSL3 and Erastin were applied. For the measurement of lipid peroxidation and cell death, 10^6^ cells were seeded in 2 mL of medium in 6-well plates and incubated overnight. Subsequently, the cells were treated with specified concentrations of RSL3, Erastin, FINO_2_, SB, TSA, and SAHA for a duration of 24 h. To detect lipid peroxidation, the cells were incubated with 2 mM C11 BODIPY 581/591 (Invitrogen, CA, USA, D3861), while for the detection of cell death they were incubated with propidium iodide (PI) (Invitrogen, CA, USA, R37169) at a concentration of 1 mg/mL in PBS. Following two washes with PBS, fluorescence-activated cell sorting (FACS) analysis was performed on the collected cells using FL1 channel for C11 BODIPY and FL2 channel for PI.

***RNA extraction, reverse transcription and real-time quantitative PCR (RT‒qPCR), mRNA stability assay, and RNA sequencing***

Total RNA was extracted from cells using TRIzol Reagent (Invitrogen, CA, USA, 15596018CN). Subsequently, cDNA synthesis was performed on the total RNA using a PrimeScript™ RT Reagent Kit (Takara Bio, Otsu, Japan, RR037A). mRNA expression levels were quantified by real-time quantitative PCR in triplicate using a SYBR Premix Ex Taq™ kit (Takara Bio, Kyoto, Japan, RR420A) and an ABI 7900HT Real Time PCR system (Applied Biosystems Life Technologies, Foster City, CA, USA). The specific primers utilized are listed in Table S2. The comparative cycle threshold (2 ΔΔCt) method was employed for data analysis.

For mRNA stability assay, the cells were treated with Actinomycin D (MedChemExpress, New Jersey, Japan, HY-17559) at a concentration of 5 μg/mL. After incubation for 0, 2, and 4 h, the cells were collected and mRNA was extracted for RT-qPCR as previously described.

For RNA-seq, 1 µg of RNA per sample was utilized as input material for RNA preparation. Sequencing libraries were generated using the NEBNext® UltraTM RNA Library Prep Kit for Illumina® (NEB, USA), and index codes were incorporated to assign sequences to each sample. The index-coded samples were clustered on a cBot Cluster Generation System using TruSeq PE Cluster Kit v3-cBot-HS (Illumia, CA, USA, PE-401-3001). After clustering, the library preparations were sequenced using an Illumina Novaseq6000 platform, generating 150 bp paired-end reads. The raw data (raw reads) in fastq format underwent initial processing through custom perl scripts. During this step, clean data (clean reads) were obtained by eliminating reads containing adapters, ploy-N sequences, or low-quality reads. Subsequently, STAR was employed to align the clean reads with the reference genome. HTSeq v0.6.0 was utilized for read counting mapped to each gene. Following that, FPKM values for each gene were calculated based on both gene length and read count information. To filter differentially expressed genes, DESeq2 algorithm was applied considering two criteria: (i) |log2FC| >1.5 and (ii) P-value <0.05. RNA-seq profiles have been deposited in GEO database under accession number GSE263463.

***CRISPR/Cas9-mediated genome editing and*** ***Western Blotting analysis***

For CRISPR/Cas9-mediated genome editing, single-guide RNAs (sgRNAs, Table S3) were designed using the website http://crispr.mit.edu and inserted into the CRISPR-V2 vector (Addgene, #52961). Lentivirus packaging and infection were conducted following previously described protocols. Following antibiotic screening (2 μg/mL puromycin), single cells were sorted into 96-well plates to obtain individual clones. Subsequently, the clones were validated for genome editing through qRT-PCR and Western blot analysis. The Western blot analysis was performed using rabbit polyclonal or monoclonal antibodies specific for the proteins: Actin, DHODH, GPX4, and Histone 3 (1:1000 dilution; Cell Signal Technology, Massachusetts, US), pan-Kla (1:1000 dilution; PTM BioLab, Hangzhou, China), FSP1, IGF2BP1 (1:1000 dilution; ProteinTech Group, Wuhan, China), ACSL4, FTO, SLC7A11, and ALKBH5 (1:1000 dilution; ABclonal, Wuhan, China). For generation of HDAC1^K412lac^ antibody, two peptides, “CSSD-(lacty) K-RIASEEE 408-419" and“CSSDKRIASEEE 408-419" were synthesized and immunized in New Zealand rabbit.

***RNA interference***

Small inhibitory RNA (siRNA) library against HDAC and pooled scrambled control siRNA were purchased from RiboBio Co., Ltd. (Guangzhou, China). Sequences of all siRNAs were provided in Table S4. siRNAs were transfected into CRC cell lines using HighGene Transfection reagent (ABclonal, Wuhan, China, RM09014P). RNA samples were collected 60 h after siRNA transfection.

***CoQ and CoQH_2_ analysis***

Cell extracts were prepared by adding 600 μl of freshly prepared, ice-cold isopropanol containing 100 μM tert-butyl-hydroquinone to the plates under ice-cold conditions. Subsequently, cell extracts were centrifuged at 13,000 rpm at 4 °C for 5 min. The supernatants were transferred to amber glass sample vials in a temperature-controlled autosampler set at 4 °C. Separation was conducted on a Thermo Scientific UltiMate 3000 HPLC system utilizing an Acquity UPLC HSS T3 column (2.1 × 100 mm, with a particle size of 1.8 μm). The HPLC parameters are as follows: Mobile phase A (acetonitrile:water (60:40, v/v) with 10 mM ammonium acetate and 0.1% acetic acid), Mobile phase B (isopropanol:acetonitrile:water (85:10:5, v/v/v) with 10 mM ammonium acetate and 0.1% acetic acid), Gradient (0 min, 40% B; 1.5 min, 40% B; 12 min, 100% B; 15 min, 100% B; 16 min, 40% B; 17 min, 40% B), Injection volume (10 μl), Column temperature (55 °C), and Flow rate (400 μl/min).

The samples were analyzed using a heated electrospray ion source in positive ionization mode on an Exactive orbitrap mass spectrometer. The instrument parameters are as follows: sheath gas flow rate of 30 (arbitrary units), auxiliary gas flow rate of 10 (arbitrary units), sweep gas flow rate of 3 (arbitrary units), spray voltage of 4 kV, capillary temperature maintained at 120 °C, heater temperature set to 500 °C, capillary voltage adjusted to 65 V, and tube lens voltage set at 100 V. The scan range was configured from 200 to 1000 m/z, employing a maximum injection time of 100 ms. A resolution of 10^6^ at a frequency rate of 1 Hz was applied along with an AGC (automatic gain control) target set at approximately one million. The acquired data underwent analysis utilizing the MAVEN software suite version number fifty-seven. Signal intensity was quantified based on Peak Area (Top), leading to the detection of both CoQ and CoQH_2_ in their respective ammonium adduct forms ([M+NH4] ^+^).

***Immunohistochemical (IHC) staining***

The paraffin-embedded sections were subjected to IHC. Deparaffinization of the sections was carried out using xylene, followed by hydration with decreasing concentrations of ethanol (100%, 90%, 80%, and 75%) for a duration of 3 min each time. Antigen retrieval was performed by microwave heating the sections in sodium citrate buffer. Subsequently, the sections were blocked with 5% BSA and incubated overnight at 4 °C with anti- FSP1 rabbit polyclonal antibody (1:100 dilution; ABclonal, Wuhan, China). Afterward, the sections were treated with horseradish peroxidase (HRP)‑conjugated rabbit secondary antibody (1:200 dilution; ProteinTech Group, Wuhan, China) for 1 h at room temperature. This was followed by 3,3′‑diaminobenzidine development (DAB Substrate Chromogen System; Merck KGaA, Darmstadt, Germany, D7304) and hematoxylin staining. Finally, fixation of the sections took place before obtaining images using an inverted microscope (Olympus IX71, Japan). The H-score was determined by applying the formula (H-score = percentage of cells exhibiting weak intensity × 1 + percentage of cells exhibiting moderate intensity × 2 + percentage of cells exhibiting strong intensity × 3) (44).

***Quantification of RNA modifications by LC /MS***

Total RNAs were isolated using TRIzol reagent (Invitrogen, CA, USA, 15596018CN). 200 ng tRNA was incubated with nuclease P1 (Merck, Darmstadt, Germany, N8630) in a 20 μL reaction mixture composed of 25 mM NaCl and 2.5 mM ZnCl_2_ at 37 °C for 2 h. Subsequently, 2.2 μL of NH_4_HCO_3_ (1 M) and alkaline phosphatase were introduced to the reaction mixture, which was then further incubated at 37 °C for 2 h. After centrifugation at 13000 rpm for 10 min at 4 °C, 10 μL of the solution was analyzed by LC/MS.

***Dot Blot assay***

Total RNAs were isolated by the Trizol reagent as described above. Then denaturation of the RNAs was achieved by heating at 95℃ for 5 min, followed by rapid chilling on ice. The RNA (200-400 ng) was spotted directly onto the positively charged nylon membrane (Beyotime, Shanghai, China, FFN08) and air-dried for 5 min. Then the membrane was crosslinked under 254 nm-UV in an Ultraviolet Crosslinker. To prevent non-specific binding, the membrane was blocked with 5% nonfat milk in TBST and then the membrane was incubated with an anti- m^6^A antibody (Millipore, Massachusetts, US, MABE1006) overnight at 4℃. HRP-conjugated anti-rabbit IgG secondary antibody was added to the membrane for 1 h at room temperature and then developed with enhanced chemiluminescence. Methylene blue (Merck KGaA, Darmstadt, Germany, M9140) staining was used to verify that an equal amount of mRNA was spotted on the membrane.

***RNA immunoprecipitation (RIP) and methylated immunoprecipitation (meRIP)***

For RIP assay, the Magna RIP Kit (Millipore, Massachusetts, USA, 17-700) was used. Cells were lysed using RIP lysate buffer and incubated with Protein A/G Beads (Millipore, Massachusetts, US, 16-663) conjugated IGF2BP1 rabbit antibody (ProteinTech Group) overnight at 4 °C. After digesting protein by proteinase K, the enriched RNA was extracted by Trizol reagent, followed by qRT-PCR analysis of the FSP1 level. For the meRIP assay, the Magna MeRIP™ m^6^A Kit (Millipore, 17-10499) was used. Total RNA was fragmented into 100nt or less for 5 min at 70 °C, followed by incubation with anti-m^6^A antibody and Magna Protein A/G Magnetic Beads overnight at 4 °C. The enriched RNA was eluted by m^6^A 5′-monophosphate sodium salt, followed by RNA extraction and qRT-PCR analysis.

***RNA pull-down assay***

The transcription of FSP1 RNA was synthesized using a Transcript Aid T7 High Yield Transcription Kit (Thermo Fisher Scientific, K0441). The RNA pull-down assay was performed using a Pierce Magnetic RNA-protein pull-down Kit (Thermo Fisher Scientific, CA, USA, 20164) following the manufacturer’s protocol. Briefly, the obtained RNA was biotinylated and incubated with streptavidin magnetic beads and RNA capture buffer for 30 min at room temperature with rotation. Subsequently, the sediment was separated using a magnetic separator and mixed with 100 µL Protein-RNA Binding Buffer, followed by incubation for 60 min at room temperature. Next, the protein from the sample mixture was extracted and combined with the biotinylated RNA mixture and SDS loading buffer. Finally, this mixture was heated for 5 min at 95 °C before being detected by silver stain and mass spectrum.

***Chromatin immunoprecipitation (CHIP) assay***

CHIP assay was performed according to the manufacturers’ instructions by using Magna ChIP Kit (Millipore, Massachusetts, US, 17-20000). Chromatin samples were immunoprecipitated with antibodies against a negative control normal rabbit IgG, H3K9ac, H3K14ac, H3K27ac, H3K56ac and H4K16ac (Abclonal, A7255, A25314, A7253, A22565, and A23091), respectively. Subsequently, IP production was performed with RT-qPCR as described above. The primers of FTO promoter were as follows: Forward: 5′- AACATGGCAGGCTCCCGTAG-3′ and reverse: 5′- GGAGAATTTCCCAGGTCCGA-3′. The primers of ALKBH5 promoter were as follows: Forward: 5′- TTGGCAATATGAGCGCACCC-3′ and reverse: 5′- CCCACTCTCTGAGGGGCTTC-3′.

***Co-immunoprecipitation (Co-IP)***

Cell lysates were pre-cleared by incubating with 20 μL protein A/G agarose (SantaCruz, Dallas, TX, USA, sc-2003). After that, the supernatant was incubated with appropriate primary antibodies at 4 °C for 3 h, followed by incubation with 40 μL protein A/G agarose at 4 °C for 30 min. The enriched proteins were separated by SDS-PAGE gel and analyzed by Western blotting.

***LC-MS for*** ***lactylation detection***

10 paired CRC and paired normal bowel tissues were collected from surgery and mixed into one CRC/normal group, respectively (Clinical features were provided in Table S5). Samples were ground with liquid nitrogen into cell powder and then treated with lysis buffer (8 M urea, 1% protease inhibitor cocktail). After sonication on ice using a high-intensity ultrasonic processor (Scientz, Ningbo, China, DNU-1020), the debris was removed by centrifugation. Subsequently, the protein solution was reduced with 5 mM dithiothreitol, alkylated with 11 mM iodoacetamide, and diluted with 100 mM TEAB. The first digestion occurred overnight using a 1:50 trypsin-to-protein mass ratio, followed by a second 4-hour digestion at a 1:100 ratio. The resulting peptides were desalted by C18 SPE column (Thermo Fisher Scientific, 60108-301). To enrich modified peptides, tryptic peptides dissolved in NETN buffer were incubated with pre-washed pan-Kla antibody beads. After bead washes, the bound peptides were eluted and then vacuum-dried. For LC-MS/MS analysis, the peptides were desalted with C18 ZipTips (Millipore, Massachusetts, US, Z720038). They were separated and subsequently analyzed using a timsTOF Pro (Bruker Daltonics) mass spectrometer in PASEF mode. The MS/MS data were processed using MaxQuant search engine (v.1.6.15.0), searching tandem mass spectra against a database with specified cleavage enzyme and mass tolerances. Modifications were also assigned, and the FDR was controlled at < 1%.

| Factors |  | Number |
| --- | --- | --- |
| pT | pT1 | 9 |
|  | pT2 | 9 |
|  | pT3 | 48 |
|  | pT4 | 13 |
| pN | N0 | 48 |
|  | N1 | 18 |
|  | N2 | 13 |
| pM | M0 | 77 |
|  | M1 | 2 |
| Age | ≤60 | 25 |
|  | ＞60 | 54 |
| Gender | Male | 54 |
|  | Female | 25 |
| Colorectal cancer cohort (n=79). | | |

**Table S1.** Clinical characteristics of CRC cohort used for IHC

**Table S2.** Primer of qRT-PCR

| Gene | Forward Primer | Reverse Primer |
| --- | --- | --- |
| FSP1 | 5′-AGACAGGGTTCGCCAAAAAGA-3 | 5′-CAGGTCTATCCCCACTACTAGC-3′ |
| SLC7A11 | 5′-TCTCCAAAGGAGGTTACCTGC-3′ | 5′-AGACTCCCCTCAGTAAAGTGAC-3′ |
| ACSL4 | 5′-CATCCCTGGAGCAGATACTCT-3′ | 5′-TCACTTAGGATTTCCCTGGTCC-3′ |
| FTO | 5′-ACTTGGCTCCCTTATCTGACC-3′ | 5′-TGTGCAGTGTGAGAAAGGCTT-3′ |
| ALKBH5 | 5′-CGGCGAAGGCTACACTTACG-3′ | 5′-CCACCAGCTTTTGGATCACCA-3′ |
| METTL3 | 5′-TTGTCTCCAACCTTCCGTAGT-3′ | 5′-CCAGATCAGAGAGGTGGTGTAG-3′ |
| METTL14  WTAP | 5′-AGTGCCGACAGCATTGGTG-3′  5′- CTTCCCAAGAAGGTTCGATTGA-3′ | 5′- GGAGCAGAGGTATCATAGGAAGC-3′  5′- TCAGACTCTCTTAGGCCAGTTAC-3′ |

**Table S3.** sgRNA and sequences of target genes

| Name | Sequence (5'-3') |
| --- | --- |
| sgFSP1 #1  sgFSP1 #2 | GAATCGGGAGCTCTGCACG  CCAGCGCTCACGGTTCATCG |
| sgIGF2BP1 #1 | CAAGATCATCTTACAAGCGG |
| sgIGF2BP1 #2 | CTCGTCCGGGCAGTCCACGA |
| sgHDAC1 #1 | GTTAACTACCCGCTCCGAGA |
| sgHDAC1 #2  sgALKBH5 #1  sgALKBH5 #2  sgFTO#1  sgFTO #2 | GAGATGTTCCAGCCTAGTG  CCTCATAGTCGCTGCGCTCG  ATAGTTGTCCCGGGACGTCA  GAAGCGCACCCCGACTGCCG  ACGGTCCCCTGGCCAGTGAA |

**Table S4.** Sequences of HDAC siRNA library

| Name | Target sequence |
| --- | --- |
| HDAC1 si1 | GCGACTGTTTGAGAACCTT |
| HDAC1 si2 | GGGATCGGTTAGGTTGCTT |
| HDAC2 si1 | CCGTAATGTTGCTCGATGT |
| HDAC2 si2 | GACCCATAACTTGCTGTTA |
| HDAC3 si1 | GAGCAACCCAGCTGAACAA |
| HDAC3 si2 | GTCCTGCATTACGGTCTCT |
| HDAC4 si1 | GGGAATGTACGACGCCAAA |
| HDAC4 si2 | TGTCGACCTCCTATAACCA |
| HDAC5 si1 | GGACTGTTATTAGCACCTT |
| HDAC5 si2 | CAACGGGAACTTCTTTCCA |
| HDAC9 si1 | AGCCACCCTCATGTTACTT |
| HDAC9 si2 | CATTAGAGGTACCCACAAA |
| HDAC6 si1 | GGCTATAACCTGACATCCA |
| HDAC6 si2 | ACACCTACGACTCAGTTTA |
| HDAC7 si1 | CTCACGTCCAGGTGATCAA |
| HDAC7 si2 | CTGCGCTATAAGCCCAAGA |
| HDAC8 si1 | GAAGCATATGCACTGCATA |
| HDAC8 si2 | GCCAGTATGGTGCATTCTT |
| HDAC11 si1 | CTCCATACTTAATCTGTTT |
| HDAC11 si2 | CACACGAGGCGCTATCTTA |
| HDAC10 si1 | GACAGTTCGACGCCATCTA |
| HDAC10 si2 | GTGGTTTCCTGAGCTGCAT |

**Table S5.** Clinical characteristics of CRC cohort used for lactylation detection

| Factors |  | Number |
| --- | --- | --- |
| pT | pT1 | 1 |
|  | pT2 | 3 |
|  | pT3 | 6 |
|  | pT4 | 0 |
| pN | N0 | 7 |
|  | N1 | 3 |
|  | N2 | 0 |
| pM | M0 | 10 |
|  | M1 | 0 |
| Age | ≤60 | 4 |
|  | ＞60 | 6 |
| Gender | Male | 5 |
|  | Female | 5 |
| Colorectal cancer cohort (n=10). | | |


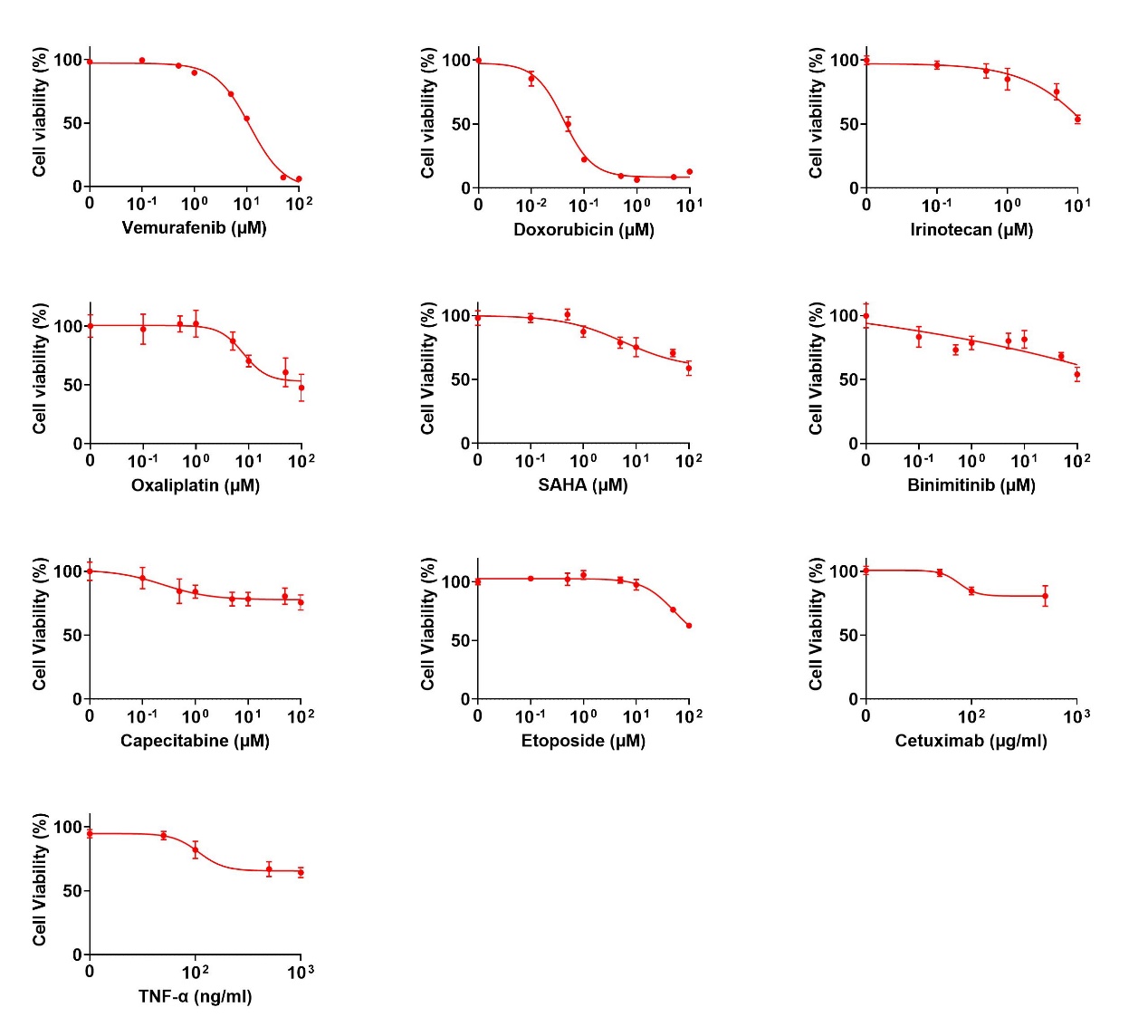


**Figure S1.** Dose-response curves of HCT116 to reagents (24 h) adopted in Figure 1D.


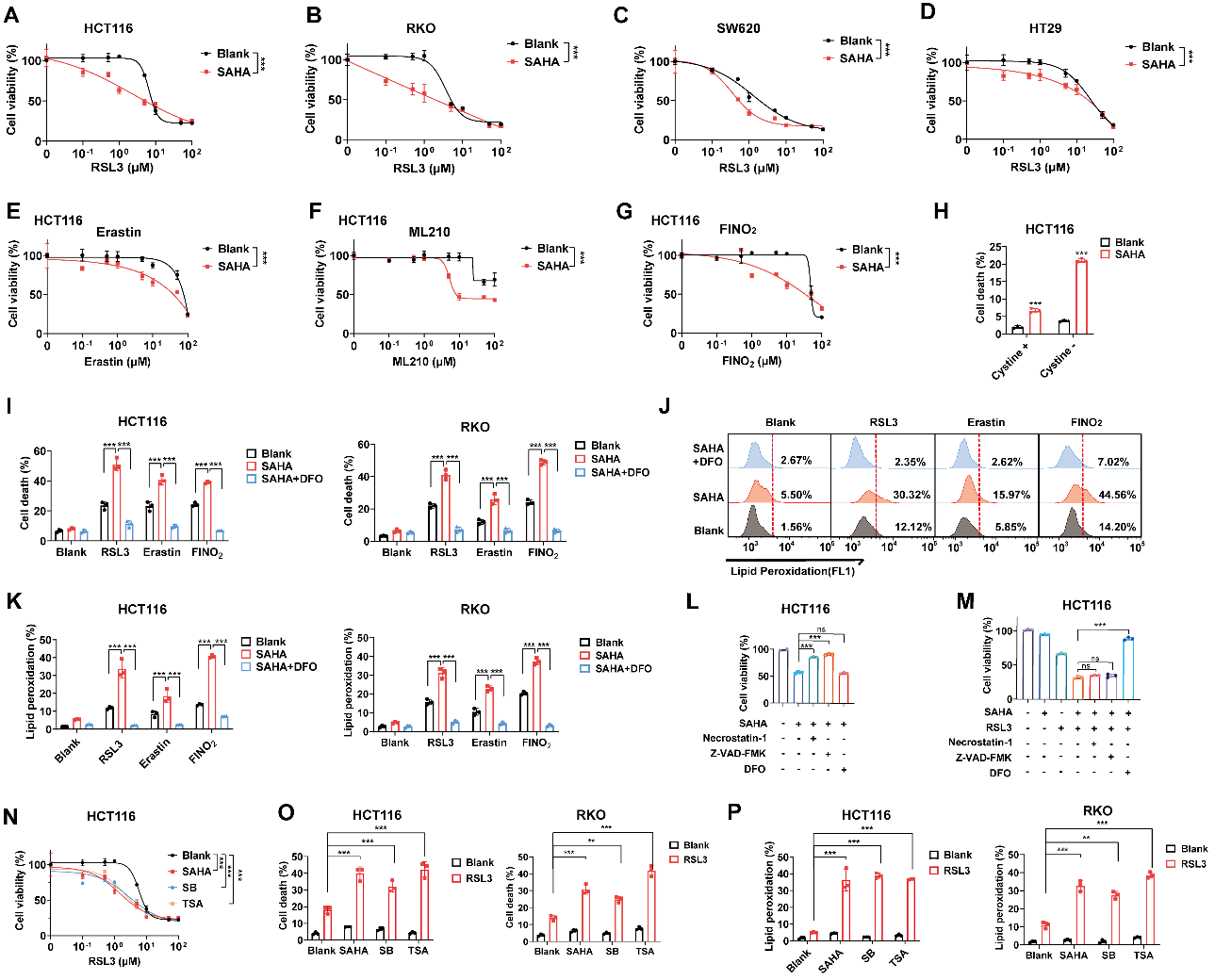


**Figure S2. HDACi confers targetable potential of colorectal cancer to ferroptosis.** (A-D) The response curve of CRC cell lines (A. HCT116. B. RKO. C. SW620. D. HT29) to multiple doses of RSL3 (0-100 μM, 24 h) with or without combining sublethal concentration of SAHA (1 μM). (E-H) The response curve of HCT116 to multiple doses of different FINs with or without combining sublethal concentration of SAHA (1 μM) for 24 h. Erastin (E), ML210 (F), FINO_2_ (G), (H) Cell death of HCT116 in control (cystine+) and cystine free (cystine -) medium with or without treating sublethal concentration of SAHA (1 μM) for 24 h. (I-K) Cell death (I, detected at 24 h) and lipid peroxidation (J and K, detected at 12 h) of CRC cells treating with FINs (RSL3: 5 µM, erastin: 40 µM, FINO_2_: 20 µM), SAHA (1 μM), and DFO (50 µM). (L) Cell viability of HCT116 treated with lethal concentration of SAHA (10 μM) and different inhibitors: necroptosis inhibitor necrostatin-1: 20 µM, apoptosis inhibitor Z-VAD-FMK: 20 µM, ferroptosis inhibitor DFO: 50 µM. (M) Cell viability of HCT116 treated with sublethal concentration of SAHA (1 μM), RSL3 (5 μM), and different inhibitors for 24 h. (N) Response curve of HCT116 to multiple doses of RSL3 (0-100 μM, 24 h) with treating different HDACi at sublethal concentrations (SAHA: 1 µM, TSA: 0.1 µM, SB: 100 µM). (O-P) Cell death (O, detected at 24 h) and lipid peroxidation (P, detected at 12 h) of CRC cells treating with RSL3 and different HDACi at sublethal concentrations. (*ns*: no significance, ***P<0.001).


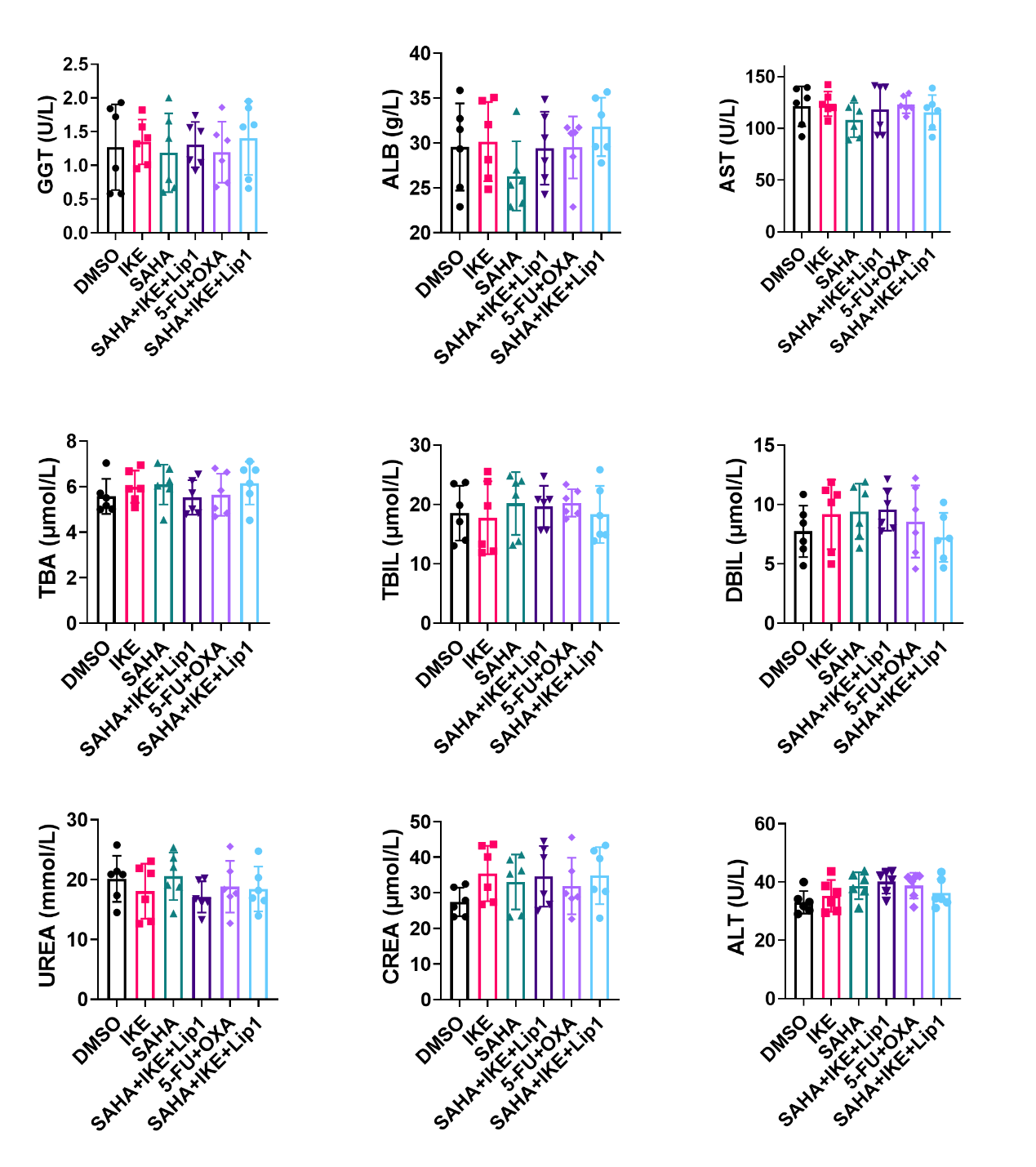


**Figure S3.** Liver/renal function test of mice in Figure 1.

**Figure S
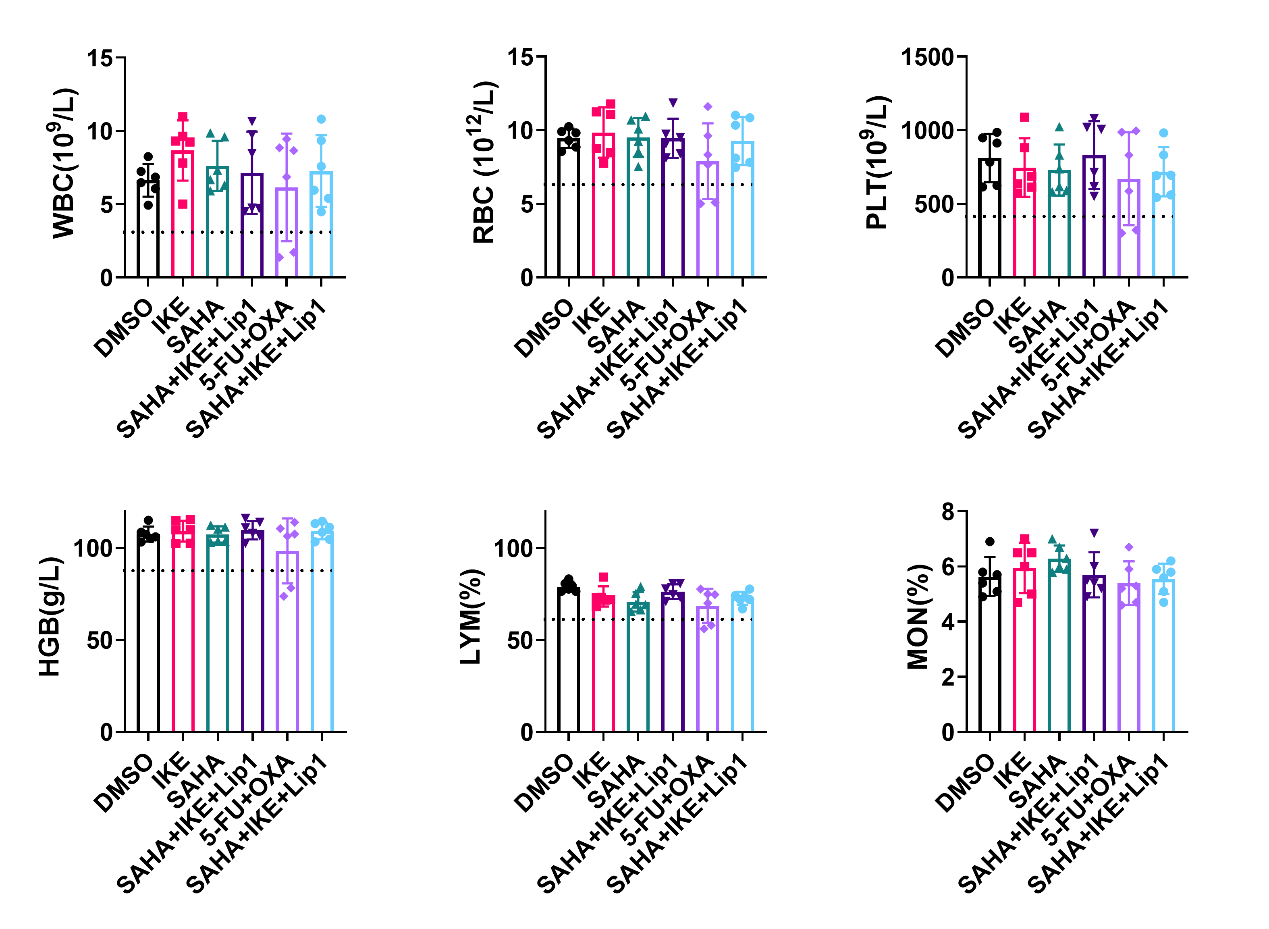
4.** Blood routine examination of mice in Figure 1.


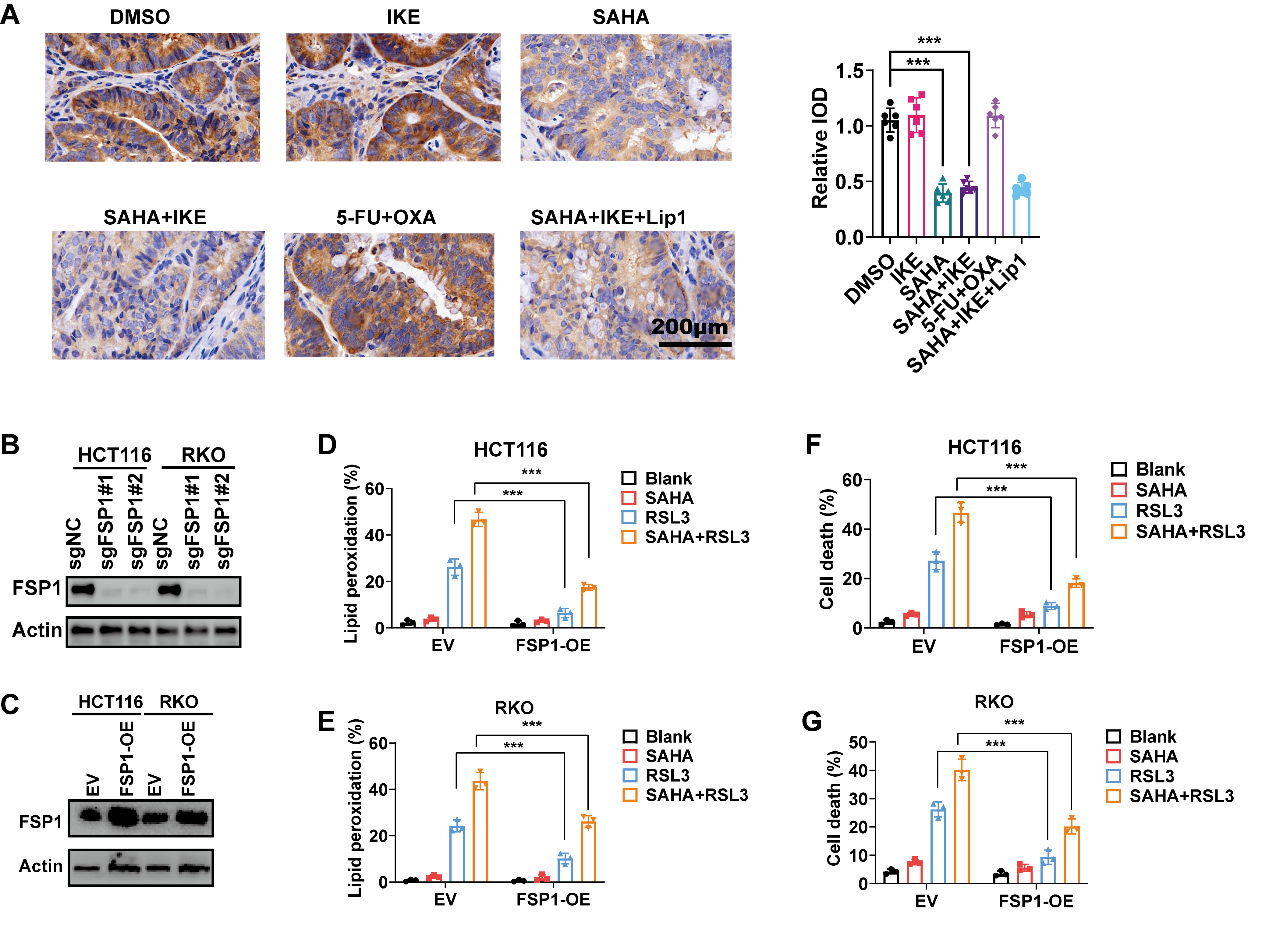


**Figure S5. HDACi drives response to ferroptosis inducer by repressing FSP1.** (A) IHC and quantification of FSP1 in AOM/DSS model mice used in Figure 1. (B-C) Depletion (B) and overexpression (C) of FSP1 in CRC cells validated by Western Blotting. (D-E) Lipid peroxidation induced by RSL3 (5 μm, 12 h) in FSP1 overexpressed (OE) HCT116 (E) and RKO (E). (F-G) Cell death induced by RSL3 (5 μm, 24 h) in FSP1 overexpressed HCT116 (F) and RKO (G). (***P<0.001). (*ns*: no significance, ***P<0.001).


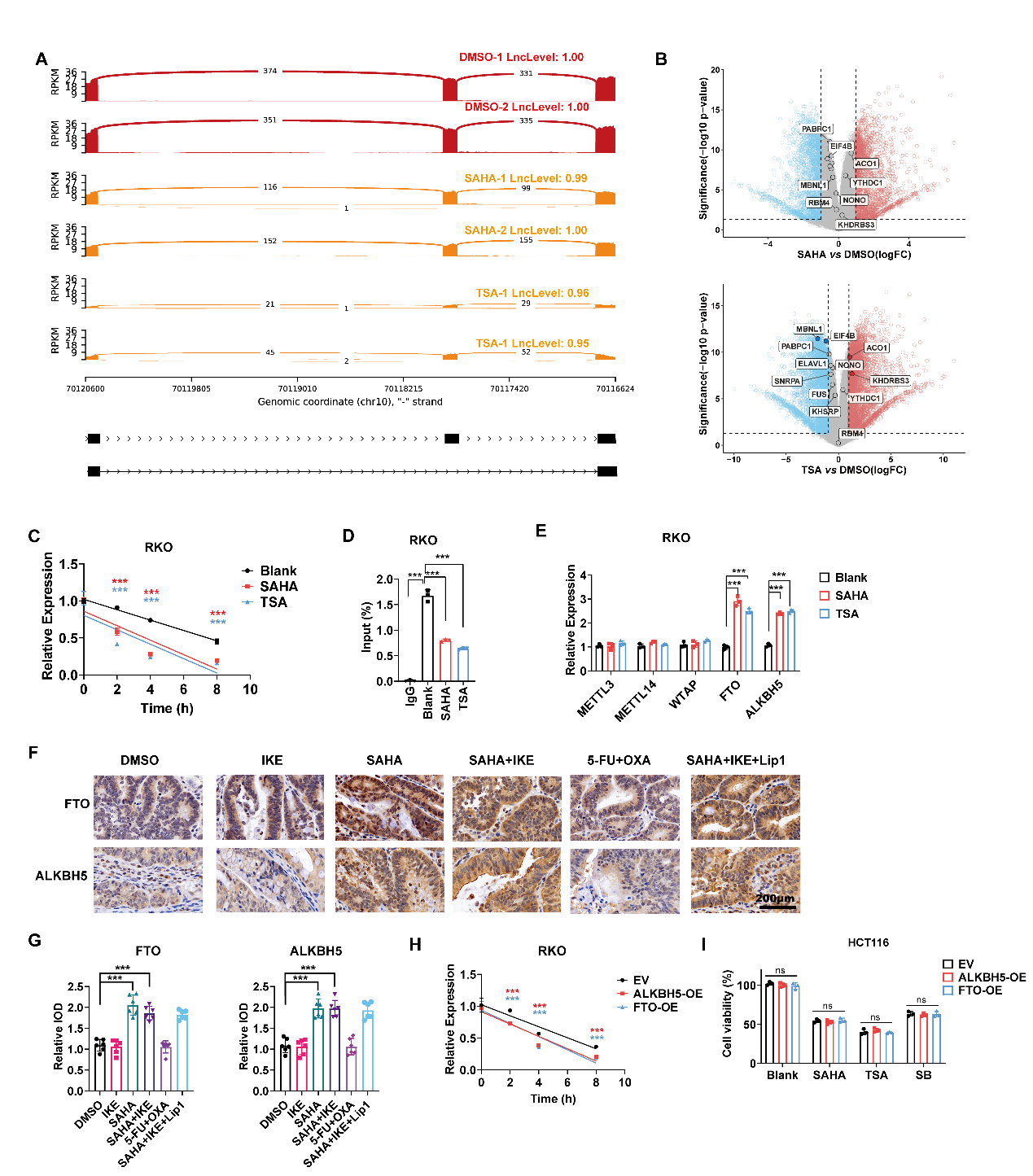
**Figure S6. HDACi accelerates mRNA degradation of FSP1 dependent on m^6^A RNA methylation.** (A) rMATS analysis of potential alternative splicing events of HCT116 with treating SAHA (1 μM) and TSA (0.1 μM) for 24 h. (B) Expression analysis of FSP1’s RNA-binding protein (RBP) in HCT116 with treating SAHA (1 μM) and TSA (0.1 μM) for 24 h. **(**C) mRNA stability of FSP1 in RKO cells treated with SAHA and TSA. (D) m^6^A level of FSP1 mRNA in RKO cells detected by meRIP-qPCR. (E) mRNA expression of m^6^A writers and erasers in RKO cells treated with SAHA and TSA. (F-G) IHC and quantification of FTO and ALKBH5 in AOM/DSS induced CRC used in Figure 1. (H) Overexpression of ALKBH5 and FTO repressed mRNA stability of FSP1 in RKO cells. (I) Cell viability of RKO cells treated with lethal dose of HDACi (SAHA: 5 µM, TSA: 0.5 µM, SB: 500 µM). (*ns*: no significance, ***P < 0.001)


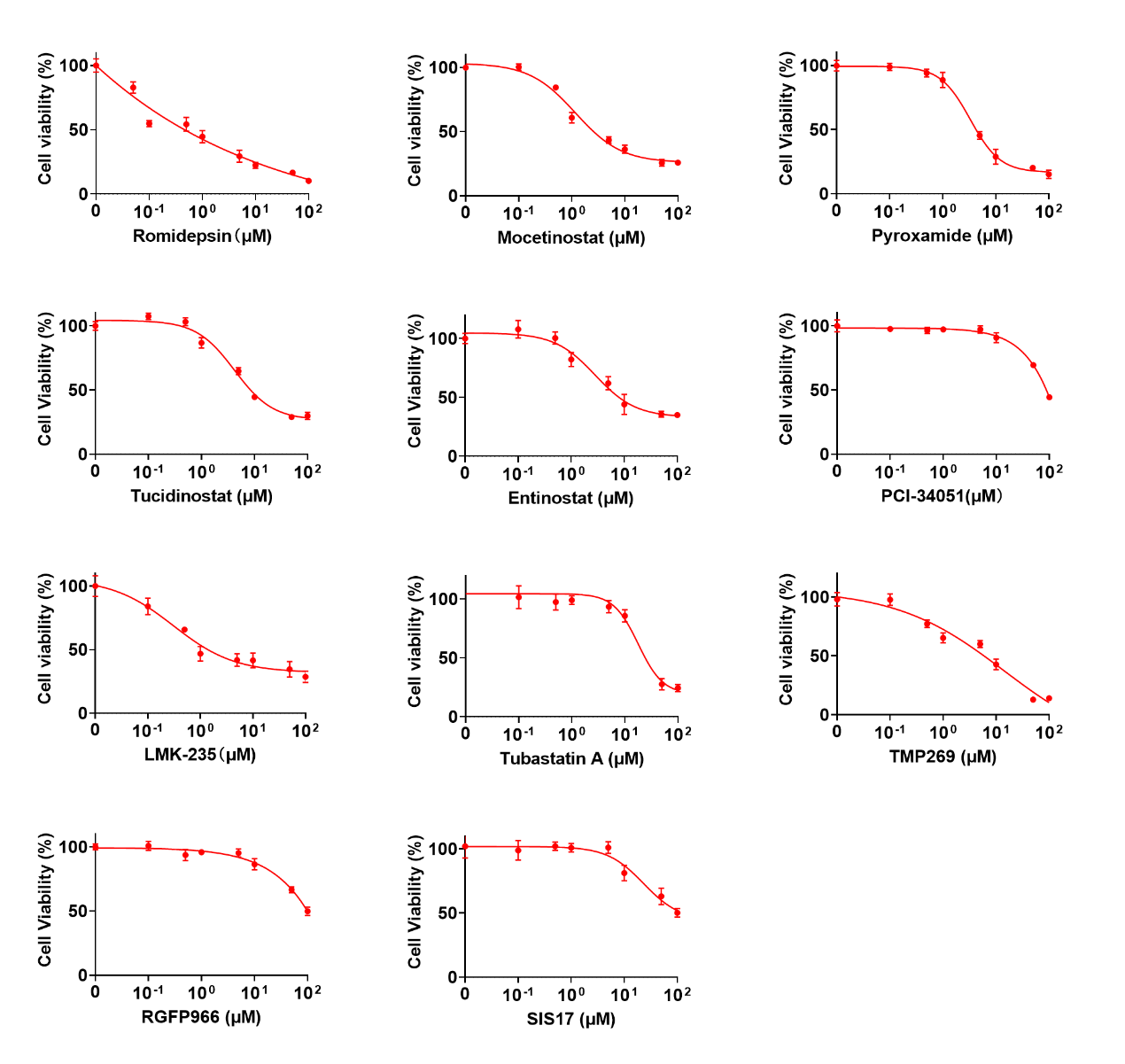


**Figure S7.** Dose-response curves (24 h) of HDACi adopted in Figure 5B.


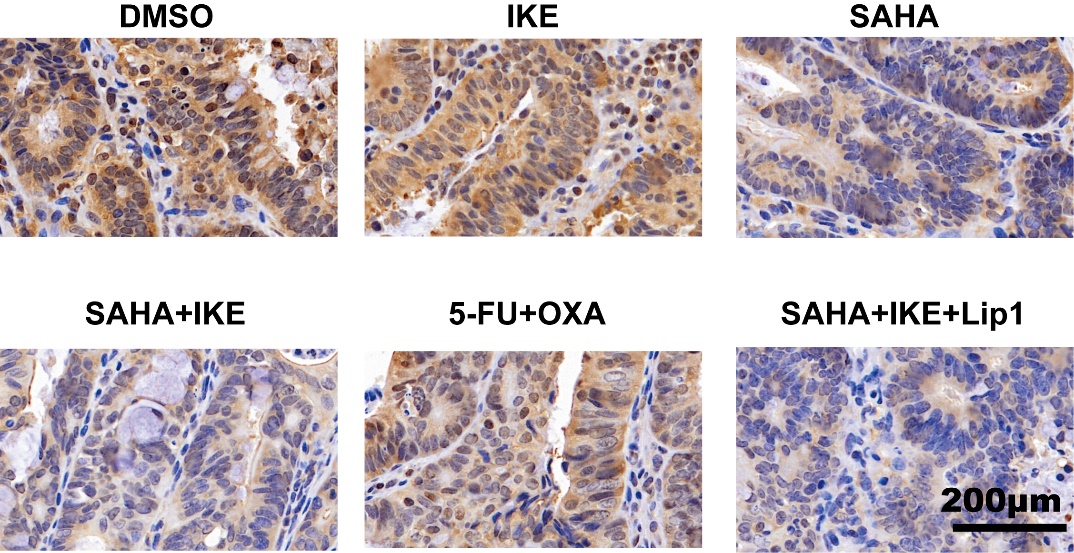


**Figure S8.** IHC staining of HDAC1^K412la^ in AOM/DSS induced CRC mice in Figure 1.
